# Epigenetic therapy reprograms M2-type tumor-associated macrophages into an M1-like phenotype by upregulating miR-7083-5p

**DOI:** 10.3389/fimmu.2022.976196

**Published:** 2022-11-22

**Authors:** Sri Murugan Poongkavithai Vadevoo, Gowri Rangaswamy Gunassekaran, Jae Do Yoo, Tae-Hwan Kwon, Keun Hur, Sehyun Chae, Byungheon Lee

**Affiliations:** ^1^ Department of Biochemistry and Cell Biology, School of Medicine, Kyungpook National University, Daegu, South Korea; ^2^ Cell & Matrix Research Institute (CMRI), Kyungpook National University, Daegu, South Korea; ^3^ Department of Biomedical Science, Graduate School, Kyungpook National University, Daegu, South Korea; ^4^ Korea Brain Research Institute (KBRI), Daegu, South Korea

**Keywords:** 5-aza-2’-deoxycytidine, epigenetic therapy, macrophage reprogramming, miR-7083-5p, trichostatin A

## Abstract

Reprogramming M2-type, pro-tumoral tumor-associated macrophages (TAMs) into M1-type, anti-tumoral macrophages is a key strategy in cancer therapy. In this study, we exploited epigenetic therapy using the DNA methylation inhibitor 5-aza-2’-deoxycytidine (5-aza-dC) and the histone deacetylation inhibitor trichostatin A (TSA), to reprogram M2-type macrophages into an M1-like phenotype. Treatment of M2-type macrophages with the combination of 5-aza-dC and TSA decreased the levels of M2 macrophage cytokines while increasing those of M1 macrophage cytokines, as compared to the use of either therapy alone. Conditioned medium of M2 macrophages treated with the combination of 5-aza-dC and TSA sensitized the tumor cells to paclitaxel. Moreover, treatment with the combination inhibited tumor growth and improved anti-tumor immunity in the tumor microenvironment. Depletion of macrophages reduced the anti-tumor growth activity of the combination therapy. Profiling of miRNAs revealed that the expression of miR-7083-5p was remarkably upregulated in M2 macrophages, following treatment with 5-aza-dC and TSA. Transfection of miR-7083-5p reprogrammed the M2-type macrophages towards an M1-like phenotype, and adoptive transfer of M2 macrophages pre-treated with miR-7083-5p into mice inhibited tumor growth. miR-7083-5p inhibited the expression of colony-stimulating factor 2 receptor alpha and CD43 as candidate targets. These results show that epigenetic therapy upon treatment with the combination of 5-aza-dC and TSA skews M2-type TAMs towards the M1-like phenotype by upregulating miR-7083-5p, which contributes to the inhibition of tumor growth.

## Introduction

Tumors need intimate support from stromal cells in the tumor microenvironment for their progression and metastasis. Tumor-associated macrophages (TAMs) are abundant stromal cells in the tumor microenvironment. M2-polarized TAMs promote tumor progression and suppress anti-tumor immunity, by secreting proteases, vascular endothelial growth factors, and immune-suppressive cytokines such as interleukin (IL)-10, IL-4, and transforming growth factor-β (TGF-β) (1–3). In contrast, M1-polarized macrophages inhibit tumor growth and secrete immune-stimulatory cytokines such as IL-6, IL-12, and interferon-γ (IFN-γ. IL-4 and lactate enriched in the tumor microenvironment promote the M2 polarization of TAMs (4, 5). In this regard, reprogramming of the M2-type, pro-tumoral TAMs into M1-type, anti-tumoral macrophages is an important strategy for cancer therapy.

Epigenetic regulation of gene expression involves DNA methylation, histone methylation or acetylation, and microRNA (miRNA)-mediated mRNA degradation. Epigenetic regulation has been shown to play a role in the differentiation, activation, and polarization of macrophages, depending on the situation (6, 7). For instance, M2 polarization is epigenetically regulated by methylation of histone H3 lysine-27 (H3K27) (8). IL-4 induces the expression of H3K27 demethylase Jumonji domain-containing 3 (JMJD3), which decreases H3K27 methylation at the promoter region of M2 marker genes and leads to the transcriptional activation of M2 macrophage marker genes (8). JMJD3 also facilitates the expression of IFN-regulatory factor 4 (IRF4), a key M2-activating transcription factor, by removing H3K27 methylation at the *irf4* gene (9). Trichostatin A (TSA), a pan-inhibitor of histone deacetylases (HDACs), inhibits the expression of IL-4-induced M2 marker arginase-1 (Arg1) in M2 macrophages (10). TSA induces the M1-like conversion of TAMs and expression of PD-L1 in tumor cells, which enhances its anti-tumor effects (11). In contrast, 5-aza-2’-deoxycytidine (5-aza-dC), an inhibitor of DNA methyltransferases (DNMTs), and knockdown of DNMT3B increases M2 marker expression in M1 macrophages (12). However, 5-aza-dC skews macrophages towards an M1 phenotype in primary mouse macrophages (13), and the effects of DNMT inhibitors on M2 macrophages have not been investigated (12). These findings suggest that the effects of DNMT inhibitors on macrophage polarization depend on the context of cell types in the tumor microenvironment.

Specific subsets of miRNAs are differentially expressed depending on the macrophage polarization state and play a role in regulating macrophage activation and polarization. For example, the levels of miR-155, miR-181, and miR-451 are higher in M1 macrophages than that of M2 macrophages (14). Inhibition of miR-155 favors the reprogramming of macrophages towards M2-type TAMs and accelerates tumor development (15). In contrast, the levels of miR-146a, miR-125a, and miR-145-5p are higher in M2 macrophages than in M1 macrophages (14). Inhibition of miR-146a decreases the expression of M2 macrophage genes in TAMs and inhibits tumor growth (16). The levels of miR-511-3p are high in M2-type TAMs, whereas overexpression of miR-511-3p inhibits M2-type gene expression in TAMs (17).

Interplay between miRNAs and epigenetic regulators has been reported. For example, miR-17-92 is controlled at the DNA methylation level by DNMT1, which is inhibited by miRNAs (18). TSA decreases the expression of the miR-106b-93-25 cluster, which is increased in prostate, gastric, and pancreatic cancers (16, 19). In contrast, TSA increases the expression of miR-7, which is reduced during cancer development and metastasis (20). However, there are few reports on the miRNAs involved in the reprogramming of M2-polarized macrophages into M1-like macrophages in response to epigenetic regulators. In this study, we exploited epigenetic therapy using 5-aza-dC, a DNA methylation inhibitor, and TSA, a histone deacetylation inhibitor, to identify the miRNAs involved in the reprogramming of M2-type TAMs into an M1-like phenotype, in response to epigenetic regulators. In the future study, the miRNAs could be exploited as an anti-cancer drug through the targeted delivery to tumors using certain types of nanoparticles (e.g., liposomes and exosomes).

## Materials and methods

### Cell culture

4T1 mouse breast tumor and LLC mouse lung tumor cell lines were purchased from American Type Culture Collection (Manassas, VA, USA). 4T1-luc cells expressing luciferase were obtained from PerkinElmer (Waltham, MA, USA). Cells were cultured in Dulbecco’s modified Eagle’s medium (DMEM; HyClone, South Logan, UT, USA) supplemented with 10% fetal bovine serum (FBS; ThermoFisher, Waltham, MA, USA) and 1% penicillin-streptomycin, at 37°C, in a humidified 5% CO_2_-containing atmosphere. Analysis of mycoplasma contamination was performed for cultured cells.

### Preparation and polarization of macrophages from bone marrow-derived monocytes and treatment of macrophages with 5-aza-dC and TSA

According to previous studies (21, 22), BMDMs were prepared and polarized into M1- and M2-type macrophages. Bone marrow cells were isolated from tibias and femurs of Balb/c mice and cultured in DMEM supplemented with 10 ng/mL of colony-stimulating factor-1 (CSF-1; Gibco, Carlsbad, CA, USA) and 10% FBS for 7 d. The culture medium was changed every other day. Next, the cells were incubated with 100 ng/mL of lipopolysaccharide and 20 ng/mL of recombinant mouse IFN-γ (R&D Systems, Minneapolis, MN, USA), for 24–48 h to achieve M1 polarization. For M2 polarization, BMDMs were incubated with 20 ng/mL of recombinant mouse IL-4 for 24–48 h. M2-polarized macrophages were treated with either 50 nM 5-aza-dC (Merck, Rahway, NJ, USA) for 72 h or 25 nM TSA (Merck) for 48 h. For treatment with the combination, M2-polarized macrophages were treated with 5-aza-dC for 24 h followed by treatment with 5-aza-dC and TSA for 48 h.

### Enzyme-linked immunosorbent assay

Concentrations of cytokines secreted into the culture medium of macrophages were measured using ELISA. One hundred microliters of cell culture medium was added to culture plates pre-coated with antibodies against IL-4 and IL-10 (R&D Systems), as well as IL-6, IL-12p40, and TGF-β (ThermoFisher Scientific) and incubated at 25°C for 1–2 h. The plates were then incubated with biotin-conjugated antibodies against the corresponding cytokines for 30 min, followed by incubation with streptavidin-conjugated horseradish peroxidase at 25°C for 30 min. The plates were incubated with stabilized chromogen followed by incubation with stop solution at 25°C for 30 min, post which the absorbance was measured at the wavelength of 450 nm using a microplate reader (ThermoFisher Scientific).

### Flow cytometric analysis

Macrophages in cultures were incubated with fluorescence-labeled antibodies against CD43, CD86, and CD206 (BioLegend, San Diego, CA, USA) and colony-stimulating factor 2 receptor alpha (CSF2RA; Santa Cruz Biotechnology, Dallas, TX, USA) for 20 min in the dark. For analysis of immune cell populations in tissues, freshly excised tumors were fragmented into several pieces. The fragmented tissues were further minced into 2–3 mm^3^ pieces and incubated with collagenase D (Roche, Basel, Switzerland) and DNase (Merck), at 37°C for 40 min. The tissue samples were filtered through a 100 µm-cell strainer (Falcon, Corning, NY, USA) to collect the digested cells. Dead cells and cellular debris were removed using Ficoll^®^ (GE Healthcare, Buckinghamshire, UK) gradient centrifugation. Collected cells were re-suspended in phosphate-buffered saline containing FBS and incubated with fluorescence dye-labeled antibodies against CD3, CD4, CD8, CD11b, CD45, CD86, CD206, F4/80, and Gr1 (BioLegend) for 30 min in the dark. Primary antibodies were diluted at a ratio of 1:100. After gating for CD45^+^ cells, the cells were analyzed for the corresponding markers. At least 10,000 events were analyzed using the FACSCalibur™ cytometer (BD Biosciences, Franklin Lakes, NJ, USA). Data were evaluated using CellQuest™ Pro (BD Biosciences).

### Quantitative reverse transcription-polymerase chain reaction

Tumor tissues (50 mg) were homogenized in QIAzol**^®^** lysis reagent (Qiagen, Hilden, Germany). Macrophages in cultures were also incubated with the lysis reagent. RNAs were isolated from the tissue and cell lysates using an miRNeasy**^®^** Mini Kit (Qiagen) and subjected to qRT-PCR. Primers for *actb, arg1*, *cd43*, *csf2ra*, *ifng*, *il-4*, *il-10*, *il-12p40*, *inos*, and *tgfb* were obtained from Bioneer (Daejeon, Korea). cDNA was synthesized using PrimerScript™ 1st strand cDNA Synthesis Kit (Takara, Shiga, Japan). qPCR using SYBR™ Green (Qiagen) was performed on a real-time cycler (Bio-Rad, Hercules, CA, USA). Relative mRNA expression levels were analyzed according to the Livak method (23).

### Cell viability assays

Tumor cells and M2-type macrophages were treated with 50 nM of 5-aza-dC and 25 nM of TSA for 24–72 h alone or in combination. To examine the effect of the conditioned medium (CM) of cultured macrophages, tumor cells were incubated with the CM of M2 macrophages in the absence or presence of 1 μM of paclitaxel for 24 h. After treatment, the cells were incubated with a fresh culture medium containing 10% Cell Counting Kit-8 solution (Dojindo, Kumamoto, Japan) for 1–4 h. Absorbance was measured at the wavelength of 450 nm and cell viability was calculated based on the absorbance.

### Animal models, whole-body bioluminescence imaging, and anti-tumor therapy

Six to eight-week-old mice were purchased from Orient Bio (Seongnam, Korea). Mice were cared for and maintained in conformance with the Guidelines of the Institutional Animal Care and Use Committee of Kyungpook National University (permission no. 2015-0017). An orthotopic breast tumor model was prepared by injecting 1×10^6^ 4T1 cells into the lower left mammary fat pad of Balb/c female mice. A syngeneic lung tumor model was prepared by injecting 1×10^6^ LLC cells into the lower right dorsal flank of C57BL/6 male mice.

Whole-body bioluminescence images were captured using an IVIS^®^ imaging system (PerkinElmer), at 10 min after intraperitoneal injection of D-luciferin (150 mg/kg body weight) into the tumor-bearing mice. Images were taken every 3 d from the tenth day of tumor inoculation, following which tumor progression of the mice was monitored. Mice were treated by intraperitoneally injecting them with 5-aza-dC (1 mg/kg body weight) and TSA (0.3 mg/kg body weight) once a day for 5 d. Tumor sizes were measured using a digital caliper, and tumor volumes were calculated using the following formula: Volume = (L × W × H)/2 (L: length, longest dimension, W: width, shorter dimension, parallel to the mouse body, and H: height, perpendicular to the length and width). At the end of the treatment, blood was collected for analysis of hematologic parameters from a few of the mice before sacrifice, and their tumor tissues were prepared for further study. The remaining mice were maintained to check the survival rates.

For depletion of CD8^+^ T cells, female Balb/c mice were intraperitoneally injected with 400 µg of monoclonal antibody against CD8α (Bio X cell, West Lebanon, NH, USA), one day before the inoculation of 4T1 tumor cells and every 5 day thereafter. The effectiveness of CD8^+^ T cell depletion was determined by means of flow cytometric analysis of CD45^+^CD3^+^CD8^+^ cells among the splenocytes or tumor-infiltrating cells after treatments.

For depletion of macrophages, Balb/c mice were intraperitoneally injected with 200 µL of clodronate liposomes (Liposoma, Amsterdam, Netherlands) 2 days prior to start of treatments. The effectiveness of macrophage depletion was determined by flow cytometric analysis of CD45+F4/80+MHCII+ cells in the splenocytes. For adoptive transfer of macrophages, mice were pre-treated with clodronate liposomes and then treated with intravenously injection of M2 macrophages (1 × 10^6^ cells) pre-treated with combination of 5-aza-dC and TSA or with miR-7083-5p (once a day, twice a week for 3 weeks).

### Microarray analysis of miRNAs

RNAs for miRNA profiling were isolated using the miRNeasy^®^ Mini Kit. Microarray analysis of miRNAs was performed using an Affymetrix GeneChip miRNA 4.0 array (Affymetrix, Santa Clara, CA, USA) by Macrogen (Seoul, Korea). Array data export, processing, and analysis were performed using Affymetrix GeneChip Command Console^®^ software. Hierarchical clustering heat map was obtained using the Euclidean method. Target genes of a miRNA were predicted using TargetScan analysis (https://www.targetscan.org/).

### Luciferase reporter assays

Oligonucleotide pairs containing the CSF2RA or CD43-3′-untranslated regions (3′-UTRs) were synthesized (Bioneer). The oligonucleotide sequences used were as follows: and 3′-TTTGATCGCCGGCGATCAGTTTAGGAGGAGATATCGGGGCGAGATC-5′ (CSF2RA) and and 3′-TTTGATCGCCGGCGATCAAGGTTAGACGAGGAATCGGGGCCAGATC-5′ (CD43). Four nanograms of the annealed oligonucleotides was incubated with 50 ng of pmirGLO dual-luciferase miRNA target expression vector (Promega, Madison, WI, USA), which was linearized by performing digestion with Pmel and Xbal restriction enzymes. After ligation, the miRNA target site was inserted into the 3′-end of the Firefly luciferase reporter gene. The humanized Renilla luciferase gene of the pmirGLO vector was used as a control reporter for the normalization of gene expression. HEK293T cells (5×10^4^ cells) were seeded into 24-well plates overnight and transfected with 2 µg of the pmirGLO plasmid containing the CSF2RA- or CD43-3′-UTR, in the absence or presence of 20 µM of miR-7083-5p or a scrambled control. Firefly luciferase activity normalized to Renilla luciferase activity was obtained 24 h after transfection of the dual-luciferase reporter expression vector using a SpectraMax^®^ L microplate reader (Molecular Devices, San Jose, CA, USA).

### Statistical analysis

Data are expressed as the mean ± standard deviation (SD). All statistical analyses were performed using Prism 6 software (GraphPad Software Inc., San Diego, CA, USA). Statistical significance was determined using one-way ANOVA followed by Tukey’s multiple comparison *post-hoc* test or two-way ANOVA followed by Bonferroni multiple comparisons *post-hoc* test.

## Results

### Epigenetic therapy upon treatment with 5-aza-dC and TSA synergistically reprograms M2-type macrophages into an M1-like phenotype

**Figure 1:**
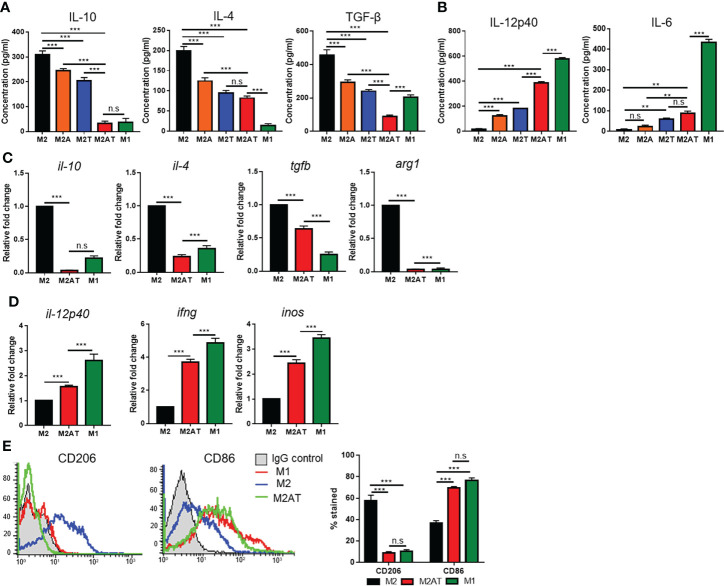
Epigenetic therapy with the combination of 5-aza-dC and TSA reprograms the M2-type macrophages towards an M1-like phenotype. **(A, B)** ELISA to quantify the M2-type **(A)** and M1-type **(B)** cytokines secreted into the culture medium after treatment of M2-type macrophages (M2) with 50 nM 5-aza-dC (M2A) and 25 nM TSA (M2T), alone or in combination (M2AT). M1-type macrophages (M1) were used as a control. **(C, D)** qRT-PCR analysis of the mRNA levels of M2-type **(C)** and M1-type **(D)** cytokines and markers after treatment of M2-type macrophages with 5-aza-dC and TSA in combination. **(E)** Flow cytometric analysis of the surface expression of CD206 and CD86 after treatment of M2-type macrophages with 5-aza-dC and TSA in combination. Data represent the mean ± SD of three separate experiments performed in triplicates. **, *P*<0.01; ***, *P*<0.001; n.s., not significant, as assessed using one-way ANOVA followed by Tukey’s multiple comparison *post-hoc* test.

To examine whether epigenetic therapy by treatment with DNA methylation and histone deacetylation inhibitors reprograms the M2-type macrophages towards the M1-like phenotype, BMDMs isolated from mouse bone marrow were treated with CSF-1 and polarized into M2-type macrophages by treatment with IL-4, and then the M2-polarized macrophages were incubated with 5-aza-dC and TSA, alone or in combination (**Supplementary Figure 1A**). The M2-polarized macrophages displayed a more spindle-like shape as compared to the M1-polarized macrophages (**Supplementary Figure 1B**). Treatment of M2-polarized macrophages with 5-aza-dC and TSA, alone or in combination, did not affect the cell viability (**Supplementary Figure 1C**). Treatment of the M2-type macrophages with either 5-aza-dC or TSA decreased the secretion of immune-suppressive M2 cytokines such as IL-10, IL-4, and TGF-β into the culture medium (**Figure 1A**), while increasing the secretion of immune-stimulatory M1 cytokines such as IL-12 and IL-6 (**Figure 1B**). Interestingly, treatment of M2 macrophages with 5-aza-dC and TSA synergistically decreased the secretion of M2 cytokines (IL-10 and TGF-β) and increased the secretion of M1 cytokines (IL-12), as compared to treatment with either 5-aza-dC or TSA alone (**Figures 1A, B**, respectively). In addition, compared to untreated M2 macrophages, treatment of M2 macrophages with the combination of 5-aza-dC and TSA significantly decreased the mRNA levels of M2 cytokines (*il-10*, *il-4*, and *tgfb*) and a marker (*arg1*) (**Figure 1C** and **Supplementary Figure 2A**), while increasing those of M1 cytokines (*il-12p40* and *ifng*) and a marker (*inos*) (**Figure 1D** and **Supplementary Figure 2B**). Moreover, treatment of M2 macrophages with the 5-aza-dC and TSA decreased the surface expression of CD206 M2 marker and increased that of CD86 M1 marker, compared to untreated M2 macrophages (**Figure 1E**). The expression of M2- and M1-type cytokines and markers in the M2 macrophage, after treatment with the combination of 5-aza-dC and TSA were skewed towards that of M1 macrophages (**Figures 1A–E**).

### CM of M2-type macrophages treated with the combination of 5-aza-dC and TSA reduces tumor cell survival and enhances sensitivity to paclitaxel

**Figure 2:**
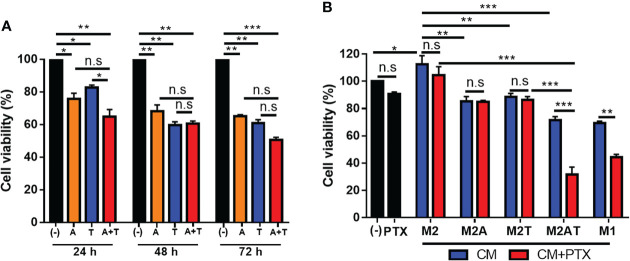
Conditioned medium (CM) of M2-type macrophages treated with the combination of 5-aza-dC and TSA inhibits tumor cell survival and sensitizes tumor cells to chemotherapy. **(A)** Cell viability of 4T1 tumor cells after treatment with 50 nM 5-aza-dC (referred as A) and 25 nM TSA(referred as T), alone or in combination (A+T), for 24–72 (h) **(B)** Cell viability of 4T1 tumor cells after incubation with the CM of M2-macrophages (M2) treated with 5-aza-dC (M2A) and TSA (M2T), alone or in combination (M2AT), in the absence (blue bars) or presence of 1 μM paclitaxel (PTX, red bars), for 24 h. Black bars represent untreated (-) and PTX-treated tumor cells in the absence of CM. The CM of M1-macrophages (M1) was used as a control. Data represent the mean ± SD of three separate experiments performed in triplicates. *, *P*<0.05; ***, *P*<0.001; n.s., not significant, as assessed using one-way ANOVA followed by Tukey’s multiple comparison *post-hoc* test **(A)** or two-way ANOVA followed by Bonferroni multiple comparisons *post-hoc* test **(B)**.

Treatment of 4T1 tumor cells with 5-aza-dC and TSA, alone or in combination, for 24–72 h reduced the cell viability (**Figure 2A**). There were no significant differences between the single and combination treatments. Next, we examined whether the CM of M2-type macrophages after the treatments caused tumor cell cytotoxicity. Treatment of 4T1 tumor cells with the CM of M2-type macrophages treated with the combination of 5-aza-dC and TSA reduced the survival of 4T1 tumor cells at higher levels than upon treatment with the CM of M2-type macrophages treated with either therapy alone (**Figure 2B**). Moreover, the CM of M2-type macrophages treated with the combination of 5-aza-dC and TSA enhanced the sensitivity of 4T1 tumor cells to paclitaxel given with the CM, resulting in about 65% cytotoxicity (**Figure 2B**). In contrast, the CM of M2-type macrophages treated with either 5-aza-dC or TSA alone did not enhance the sensitivity of 4T1 tumor cells to paclitaxel (**Figure 2B**). The CM of M1-type macrophages used as a control also reduced the viability of the tumor cells. The CM of untreated M2 macrophages rather increased the tumor cell survival (**Figure 2B**). These results suggested that, like the M1-type macrophages, M2-type macrophages treated with the combination of 5-aza-dC and TSA secrete active inflammatory or anti-tumoral factors.

### Epigenetic therapy upon treatment with 5-aza-dC and TSA synergistically inhibits tumor growth in mice and induces M2 reprogramming in the tumor microenvironment

**Figure 3:**
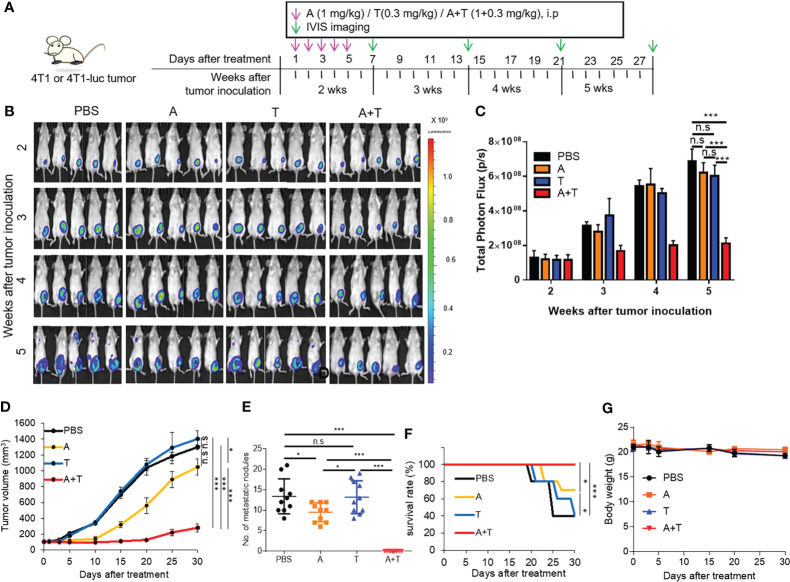
Epigenetic therapy with 5-aza-dC and TSA synergistically inhibits breast tumor growth in mice. **(A)** Experimental schemes of the tumor therapy. Mice bearing 4T1 or 4T1-luc tumors (approximately 100–150 mm^3^ in size) in the left and lower mammary gland were treated with 5-aza-dC and TSA, alone or in combination (1 mg/kg body weight 5-aza-dC and 0.3 mg/kg body weight TSA, once a day, for 5 d), at 2 weeks after tumor inoculation. **(B)** Whole-body bioluminescence images of mice bearing 4T1-luc tumors were taken 10 min after injection of D-luciferin into the mice after treatments. Scale bar represents the intensity of luminescence. **(C)** Quantitation of the total photon flux (the number of photons per second, p/s) obtained upon whole-body imaging. Data have been presented as the mean ± S.D. ***, *P*<0.001; n.s., not significant (*n*=5 per group), as assessed using two-way ANOVA. **(D–G)** Tumor volumes **(D)**, body weight **(E)**, number of metastatic nodules in the lung **(F)**, and survival rates **(G)** after treatments. Data have been presented as the mean ± S.D. *, *P*<0.05; ***, *P*<0.001; n.s., not significant (*n*=10 mice per group), as assessed using one-way ANOVA followed by Tukey’s multiple comparison *post-hoc* test.

An orthotopic breast tumor was established by inoculating 4T1 mouse syngeneic breast tumor cells into the fat pad of the mammary gland of mice. Tumor-bearing mice were treated with 5-aza-dC and TSA and monitored using whole-body luminescence imaging (**Figure 3A**). Treatment with 5-aza-dC and TSA synergistically inhibited primary tumor growth (**Figures 3B–D**) as well as lung metastasis (**Figure 3E**), and increased mouse survival rate (**Figure 3F**) in 4T1 tumor-bearing mice, as compared to treatment with either 5-aza-dC or TSA alone and that with the saline control. Treatment with 5-aza-dC alone inhibited tumor growth and metastasis and increased the survival rate of 4T1 tumor-bearing mice to a lesser degree than upon treatment with the combination, while treatment with TSA alone showed no significant effects on these parameters (**Figures 3B–F**). These treatments did not show significant changes in the body weight (**Figure 3G**) and hematologic parameters including the number of white blood cells (**Supplementary Figure 3**).

**Figure 4:**
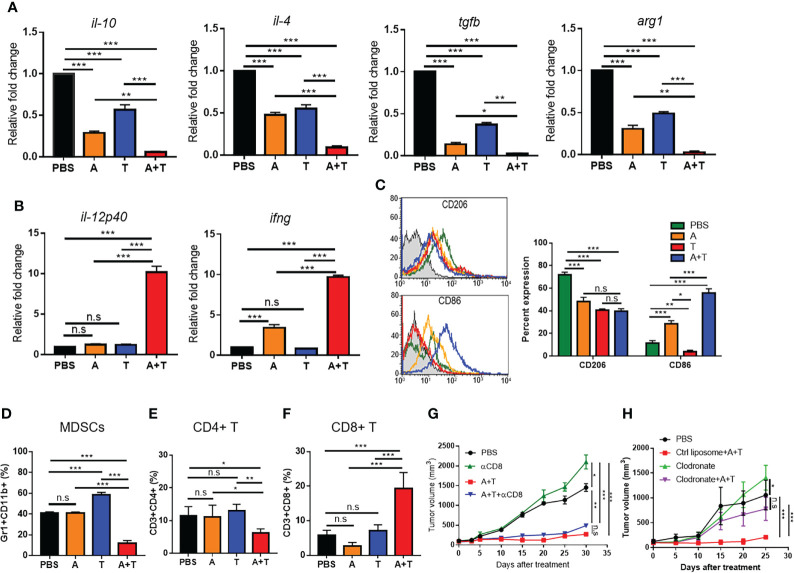
Epigenetic therapy with the combination of 5-aza-dC and TSA promotes reprogramming of M2-type TAMs into the M1-like phenotype in the tumor microenvironment. **(A, B)** qRT-PCR analysis of the relative mRNA levels of M2- **(A)** and M1-type **(B)** cytokines and markers in tumor tissues after treatment of 4T1 tumor-bearing mice with 5-aza-dC (referred as A) and TSA (referred as T), alone or in combination (A+T). (**C–F)** Flow cytometric analysis of the population of CD206^+^ M2 macrophages and CD86^+^ M1 macrophages **(C)**, CD11b^+^Gr1^+^ MDSCs **(D)**, CD4^+^ T cells **(E)**, and CD8^+^ T cells **(F)** in the tumor tissues after treatments. **(G, H)** Tumor volumes after treatments in the absence or presence of CD8^+^ T cell depletion **(G)** and macrophage depletion using clodronate **(H)**. Control (Ctrl) liposomes were used as control for clodronate. Data represent the mean ± SD of three separate experiments performed in triplicates **(A–F)** and three mice (*n*=3) per group **(G, H)**. *, *P*<0.05; **, *P*<0.01; ***, *P*<0.001; n.s., not significant, as assessed using one-way ANOVA followed by Tukey’s multiple comparison *post-hoc* test.

To examine whether treatment with the combination of 5-aza-dC and TSA promoted M2 reprogramming in the tumor microenvironment, the levels of macrophage cytokines/markers and the immune cell population in tumor tissues were analyzed. Treatment with 5-aza-dC and TSA synergistically decreased the mRNA levels of M2 cytokines (*il-10*, *il-4*, and *tgfb*) and a marker (*arg1*) in tumor tissues (**Figure 4A** and **Supplementary Figure 4A**), while increasing those of M1 cytokines (*il-12p40* and *ifng*) in tumor tissues (**Figure 4B** and **Supplementary Figure 4B**), as compared to either therapy alone and in the saline-treated control. In addition to that of cytokines, the population of CD206^+^ M2 macrophages (**Figure 4C**), myeloid derived suppressor cells (MDSCs) (**Figure 4D**), and CD4^+^ T cells (**Figure 4E**) reduced more efficiently after treatment with the combination of 5-aza-dC and TSA, while increasing the population of CD86^+^ M1 macrophages (**Figure 4C**) and CD8^+^ T cells (**Figure 4F**) among the tumor-infiltrating leukocytes in the 4T1 tumor tissues, as compared to those upon treatment with 5-aza-dC or TSA alone and those in the saline-treated group. As the number of CD8^+^ T cells was considerably higher in the 4T1 tumor tissues after the combination treatment, we examined whether the increase in the number of CD8^+^ T cells contributes to the anti-tumor growth activity. Pre-treatment of tumor-bearing mice with an anti-CD8 neutralizing antibody almost depleted the population of CD8^+^ T cells (**Supplementary Figures 5A, B**). However, depletion of CD8^+^ T cells did not significantly decrease the anti-tumor growth activity after the combination treatment (**Figure 4G**). In contrast, depletion of macrophages by pre-treating mice with clodronate almost reduced the anti-tumor growth activity by the combination treatment (**Figure 4H** and **Supplementary Figures 5C, D**). These results suggested that a decrease in the M2 macrophage population but not an increase in the CD8^+^ T cell population is the primary factor for the therapeutic effect of the treatment with the combination of 5-aza-dC and TSA.

Treatment with the combination of 5-aza-dC and TSA significantly inhibited the growth of the subcutaneous LLC mouse syngeneic lung tumor (**Supplementary Figure 6A**) and increased the mouse survival rate (**Supplementary Figure 6B**), as compared to treatment with either 5-aza-dC or TSA alone and saline-treated control. Similarly to 4T1 breast tumor, in the LLC lung tumor as well, treatment with 5-aza-dC and TSA synergistically decreased the levels of M2 cytokines and markers (**Supplementary Figure 6C**), while increasing those of M1 cytokines (**Supplementary Figure 6D**), as compared to treatment with either therapy alone. In addition, it decreased the population of CD206^+^ M2 macrophages (**Supplementary Figure 6E**), MDSCs (**Supplementary Figure 6F**), and CD4^+^ T cells (**Supplementary Figure 6G**), while increasing that of CD86^+^ M1 macrophages (**Supplementary Figure 6E**) and CD8^+^ T cells (**Supplementary Figure 6H**), as compared to treatment with either therapy alone. These results showed that the combination of 5-aza-dC and TSA efficiently reprograms M2-type TAMs into an M1-like phenotype and leads to an increase in the anti-tumor immunity in the tumor microenvironment, as well as inhibition of tumor growth.

### miR-7083-5p is involved in the reprogramming of M2-type macrophages to an M1-like phenotype upon treatment with the combination of 5-aza-dC and TSA

**Figure 5:**
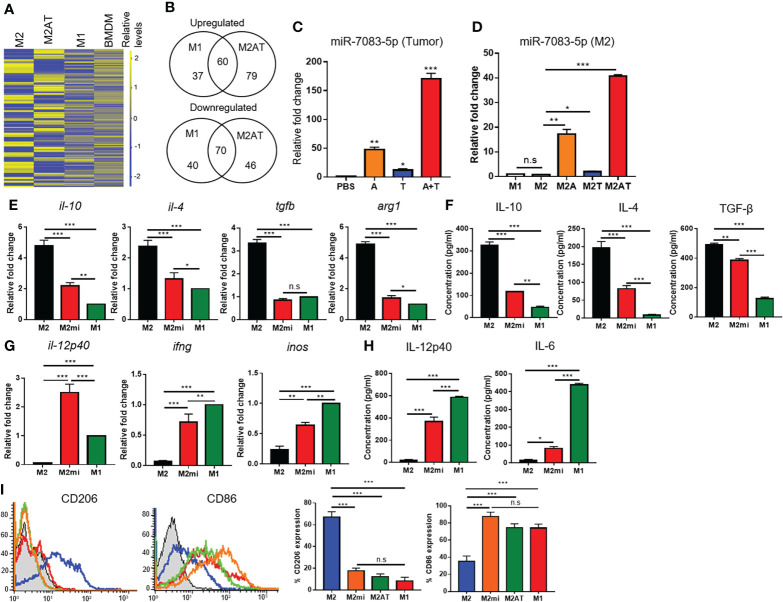
miR-7083-5p is involved in the reprogramming of M2-type macrophage into an M1-like phenotype upon treatment with the combination of 5-aza-dC and TSA. **(A)** Hierarchical clustering heat map showing the relative levels of miRNAs in M2-type macrophages (M2) after treatment with the combination of 5-aza-dC and TSA (M2AT). M1 macrophages (M1) and bone marrow-derived monocytes (BMDMs) were used as controls. **(B)** Venn diagrams showing the number of upregulated and downregulated miRNAs in M2AT and M1, as compared to those in M2. **(C, D)** qRT-PCR analysis of the relative levels of miR-7083-5p in 4T1 tumor tissues, after treatments with 5-aza-dC (referred as A) and TSA (referred as T), alone or in combination (A+T), **(C)** and in M2 macrophages, after treatments with 5-aza-dC (M2A) and TSA (M2T), alone or in combination **(D)**. **(E, G)** qRT-PCR analysis of the relative mRNA levels of M2- **(E)** and M1-type **(G)** cytokines and markers in M2 macrophages after transfection of miR-7083-5p (M2mi). **(F, H)** ELISA of the concentrations of M2- **(F)** and M1-type **(H)** cytokines secreted into the culture medium of M2 macrophages after transfection of miR-7083-5p. **(I)** Flow cytometric analysis of CD206 and CD86 expression levels in M2mi and M2AT. *, *P*<0.05; **, *P*<0.01; ***, *P*<0.001; n.s., not significant (*n*=3 per group), as assessed using one-way ANOVA. Data represent the mean ± SD of three separate experiments performed in triplicates. *, *P*<0.05; **, *P*<0.01; ***, *P*<0.001; n.s., not significant, as assessed using one-way ANOVA followed by Tukey’s multiple comparison *post-hoc* test.

To identify miRNAs that are involved in the M2 to M1 reprogramming of macrophages upon treatment with the combination of 5-aza-dC and TSA, we analyzed miRNA profiles in M2-type macrophages, before and after the treatments. Hierarchical clustering heat map showed the upregulation and downregulation of miRNAs in M2-type macrophages, after treatment with the combination of 5-aza-dC and TSA (**Figure 5A**). When treated with 5-aza-dC and TSA, the levels of 139 miRNAs were upregulated in the M2-type macrophages, as compared to those in the untreated M2 macrophages (**Figure 5B**). In addition, the levels of 97 miRNAs were higher in the M1-type macrophages than in the untreated M2 macrophages, and 60 of these miRNAs overlapped with the miRNAs found in M2 macrophages upon treatment with the combination of 5-aza-dC and TSA (**Figure 5B**). In contrast, the levels of 116 miRNAs were downregulated in the M2-type macrophages treated with 5-aza-dC and TSA in combination than in the untreated M2 macrophages (**Figure 5B**). The levels of 110 miRNAs were lower in the M1-type macrophages than in the untreated M2 macrophages, and 70 of these overlapped with the miRNAs found in M2 macrophages upon treatment with 5-aza-dC and TSA (**Figure 5B**). The miRNAs whose levels were upregulated and downregulated by more than three-fold in M2 macrophages treated with the combination of 5-aza-dC and TSA and in M1 macrophages, as compared to those in untreated M2 macrophages, are listed in **Supplementary Tables 1**, **2**, respectively. Among the upregulated miRNAs, miR-7083-5p level was remarkably (approximately 36-fold) higher in M2 macrophages treated with the combination of 5-aza-dC and TSA (**Supplementary Table 1**), whereas, it was not upregulated in M1 macrophages (**Supplementary Table 2**), as compared to that in untreated M2 macrophages. In contrast, the expression of certain miRNAs such as miR-7043-5p and miR-184-3p was upregulated both in the M2 macrophages treated with the combination of 5-aza-dC and TSA (**Supplementary Table 1**) as well as in M1 macrophages (**Supplementary Table 2**), as compared to that in untreated M2 macrophages. qRT-PCR analysis showed the upregulation of miR-7083-5p in 4T1 tumor tissues (**Figure 5C** and **Supplementary Figure 7A**) as well as M2-type macrophages in culture (**Figure 5D** and **Supplementary Figure 7B**) after treatment with 5-aza-dC and TSA. Transfection of M2 macrophages with miR-7083-5p decreased the mRNA levels of M2 cytokines and markers (**Figure 5E** and **Supplementary Figure 7C**) and secretion of M2 cytokines into the culture medium (**Figure 5F**). In contrast, upregulation of miR-7083-5p in M2 macrophages increased the mRNA levels of M1 cytokines and markers (**Figure 5G** and **Supplementary Figure 7D**) and secretion of M1 cytokines into the culture medium (**Figure 5H**). Upregulation of miR-7083-5p in M2 macrophages, as well as treatment of M2 macrophages with 5-aza-dC and TSA, decreased the surface expression of CD206 M2 marker, while increasing that of CD86 M1 marker (**Figure 5I**).

**Figure 6:**
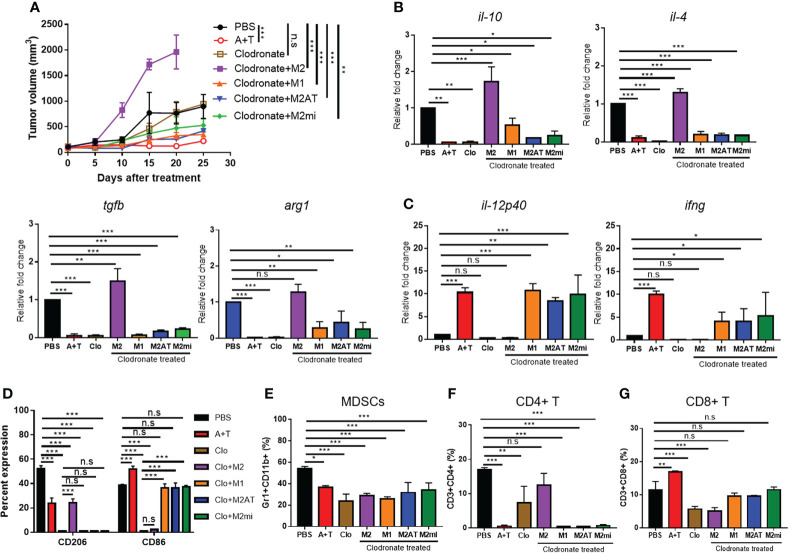
Adoptive transfer of M2 macrophages pre-treated with miR-7083-5p or combination of 5-aza-dC and TSA reduces tumor growth. **(A)** Tumor volumes after treatment of 4T1 tumor-bearing mice with clodronate and subsequent adoptive transfer of M2 macrophages pre-treated with miR-7083-5p (M2mi) or combination of 5-aza-dC and TSA (M2AT). M1 and M2 macrophages were included as control. **(B, C)** qRT-PCR analysis of the relative mRNA levels of M2- **(B)** and M1-type **(C)** cytokines and markers in tumor tissues after treatments. **(D–G)** Flow cytometric analysis of the population of CD206^+^ M2 macrophages and CD86^+^ M1 macrophages **(D)**, CD11b^+^Gr1^+^ MDSCs **(E)**, CD4^+^ T cells **(F)**, and CD8^+^ T cells **(G)** in the tumor tissues after treatments. A+T, combined treatment with 5-aza-dC and TSA. Clo, clodronate. Data represent the mean ± SD. *, *P*<0.05; **, *P*<0.01; ***, *P*<0.001; n.s., not significant (*n*=3 mice per group), as assessed using one-way ANOVA followed by Tukey’s multiple comparison *post-hoc* test.

To further examine whether miR-7083-5p is involved in the M2 macrophage reprogramming, we performed adoptive transfer of M2 macrophages pre-treated with miR-7083-5p or combination of 5-aza-dC and TSA into mice following treatment with clodronate. The adoptive transfer of pre-treated M2 macrophages inhibited the tumor growth while that of untreated M2 macrophages enhanced tumor growth compared to saline-treated control (**Figure 6A**). The adoptive transfer of pre-treated M2 macrophages decreased the levels of M2 cytokines (**Figure 6B** and **Supplementary Figure 8A**) and CD206 (**Figure 6D**) and the population of MDSCs (**Figure 6E**) and CD4+ T cells (**Figure 6F**), with no significant changes in that of CD8+ T cells (**Figure 6G**), compared to saline-treated control. In contrast, the adoptive transfer increased the levels of M1 cytokines (**Figure 6C** and **Supplementary Figure 8B**) and CD86 (**Figure 6D**) compared to saline-treated control.

### CSF2RA and CD43 are candidate targets of miR-7083-5p involved in the M2- to M1-type macrophage reprogramming

**Figure 7:**
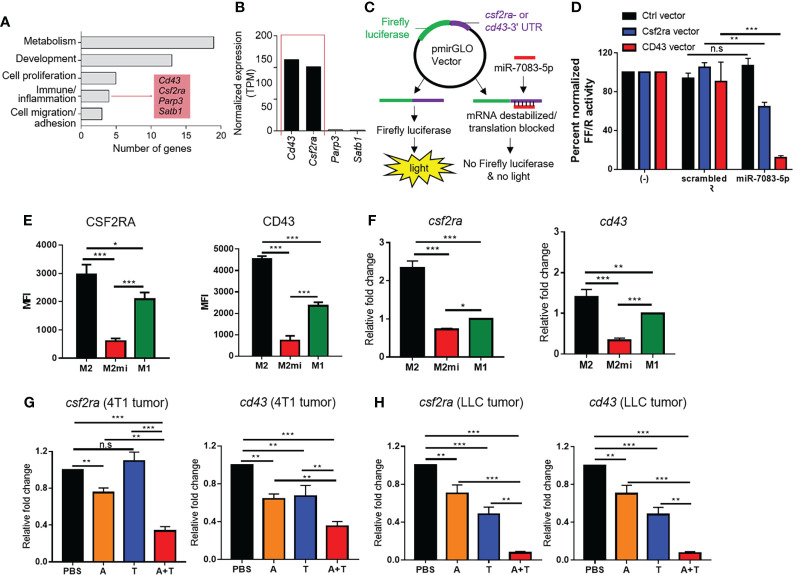
CSF2RA and CD43 are downregulated by miR-7083-5p as candidate targets. **(A)** Functional categorization of 33 predicted targets of miR-7083-5p. Functional enrichment analysis were performed using DAVID software. **(B)** Normalized expression levels of the four immune/inflammatory response-related genes from the Human Protein Atlas. **(C)** Experimental schemes of reporter assays using pmirGLO dual-luciferase miRNA target expression vector inserted with CSF2RA- or CD43-3′-UTR and a control (Ctrl) vector. **(D)** Firefly luciferase activity normalized to Renilla luciferase activity (FF/R) of HEK293T cells transfected with the pmirGLO luciferase reporter in the absence or presence of miR-7083-5p or a scrambled control. **(E)** Flow cytometric analysis of CSF2RA and CD43 expression levels in M2 macrophages (M2) after transfection with miR-7083-5p (M2mi). M1 macrophages (M1) were used as control. **(F)** qRT-PCR analysis of relative mRNA levels of *csf2ra* and *cd43* in M2 macrophages after transfection with miR-7083-5p. **(G, H)** qRT-PCR analysis of the relative mRNA levels of *csf2ra* and *cd43* in 4T1 **(G)** and LLC **(H)** tumor tissues after treatments with 5-aza-dC (referred as A) and TSA (referred as T), alone or in combination (A+T). Data have been presented as the mean ± S.D. of three separate experiments performed in five replicates. *, *P*<0.05; **, *P*<0.01; ***, *P*<0.001; n.s., not significant, as assessed using two-way ANOVA followed by Bonferroni multiple comparisons *post-hoc* test **(B)** and one-way ANOVA followed by Tukey’s multiple comparison *post-hoc* test **(C–F)**.

TargetScan database analysis was carried out to predict candidate target genes of miR-7083-5p (**Supplementary Table 3**). Functional enrichment analysis of the 33 predicted genes using DAVID software (24) indicated four genes (*cd43*, *csf2ra*, *parp3*, and *satb1*) involved in immune/inflammatory response (**Figure 7A**). Of the four genes, we further focused on the genes with high expression in the monocytes/macrophages from The Human Protein Atlas (25) and selected two genes, *cd43* and *csf2ra* (**Figure 7B**). CSF2RA, also known as granulocyte-macrophage colony-stimulating factor receptor alpha, and CD43, also known as sialophorin or leukocyte marker, are expressed in macrophages and are involved in tumor progression (26–31). To examine the targeting of the mRNAs of *csf2ra* and *cd43* genes by miR-7083-5p, HEK293T cells were transfected with a luciferase reporter vector inserted with *csf2ra-* or *cd43*-3′-UTR, in the absence or presence of miR-7083-5p or a scrambled control (**Figure 7C**). Transfection of miR-7083-5p reduced both CSF2RA and CD43 luciferase reporter activities, as compared to transfection with a scrambled control in HEK293T cells (**Figure 7D**). To further examine the role for miR-7083-5p in the regulation of CSF2RA and CD43 expression, M2 macrophages were transfected with miR-7083-5p. Flow cytometry and qRT-PCR analysis showed that the upregulation of miR-7083-5p reduced the protein (**Figure 7E**) and mRNA (**Figure 7F** and **Supplementary Figure 9A**) levels of CSF2RA and CD43 in the transfected M2 macrophages, as compared to those in the M2 and M1 macrophages). Moreover, the mRNA levels of *csf2ra* and *cd43* were significantly lower in the tumor tissues of mice bearing 4T1 (**Figure 7G** and **Supplementary Figure 9B**) and LLC (**Figure 7H** and **Supplementary Figure 9C**) tumors after treatment with the combination of 5-aza-dC and TSA than those seen upon either therapy alone.

## Discussion

This study showed that epigenetic therapy with the combination of 5-aza-dC and TSA repolarized the M2-type TAMs towards M1-like macrophages in the tumor microenvironment by upregulating miR-7083-5p expression. Epigenetic therapy-induced macrophage repolarization contributes to the improvement of anti-tumor immunity, including a decrease in the expression of immune-suppressive cytokines and MDSC population and an increase in the expression of immune-stimulatory cytokines and CD8^+^ T cell population, leading to the inhibition of tumor growth in mouse models of breast and lung tumors. In addition, this study showed that the candidate targets involved in M2 to M1 repolarization, CSF2RA and CD43, are downregulated by miR-7083-5p in M2 macrophages. This study is the first to show that miR-7083-5p plays a role in macrophage polarization, by combined inhibition of DNA methylation and histone deacetylation, and suggests miR-7083-5p as a new tool for reprogramming TAMs for cancer immunotherapy. However, given that certain types of tumors (e.g., colitis-associated colon cancer) are driven by inflammation, there may be limitations to inhibiting tumor growth by reprogramming M2 macrophages to M1-like phenotype and inducing inflammation.

Combined or simultaneous inhibition of DNA methylation and histone deacetylation regulates diverse cell types, including tumor cells, MDSCs, and TAMs, in the tumor microenvironment. For instance, simultaneous inhibition of DNA methylation by 5-aza-dC and histone deacetylation by 4-phenylbutyryl acid in tumor cells upregulates miR-127 expression and downregulates the expression of proto-oncogene *bcl6*, thereby highlighting miR-127 as a tumor suppressor (32). Treatment with the combination of low doses of 5-aza-dC and the HDAC inhibitor entinostat (0.5 mg/kg body weight per day and 5 mg/kg body weight per day, respectively) skews monocytic MDSCs towards a lung interstitial macrophage-like phenotype in the lung pre-metastatic microenvironment and contributes to the disruption of pre-metastatic niches (33). In the same context, we showed that treatment with 5-aza-dC and TSA (1 mg/kg body weight per day and 0.3 mg/kg body weight per day, respectively) synergistically promoted the reprogramming of M2-type TAMs into an M1-like phenotype, through upregulation of miR-7083-5p expression in M2 macrophages. Interestingly, miR-7083-5p was upregulated only in M2 macrophages treated with 5-aza-dC and TSA in combination but not in M1 macrophages, unlike miR-7043-5p and miR-184-3p, which were highly expressed in both cell types. In addition, the CM of M2 macrophages treated with the combination of 5-aza-dC and TSA exerted higher levels of cytotoxicity in tumor cells and, more importantly, sensitized tumor cells to paclitaxel, as compared to that treated with either therapy alone. These findings suggest a combination of chemotherapy with epigenetic regulators and paclitaxel for the management of cancer in the clinic.

Several factors involved in macrophage recruitment and polarization have been reported. CSF-1, also known as macrophage colony-stimulating factor, regulates macrophage survival and function through the downstream transcription factor ETS2 (34). Genetic depletion of CSF-1 reduces TAM levels and the incidence of lung metastasis (34). Depletion of ETS2 in TAMs reduces metastasis in mouse breast tumor models (35). The CSF-1 and ETS2 pathways upregulate certain oncogenic miRNAs, including miR-223, miR-21, miR-29a, and miR-142-3p, which promote the pro-tumoral functions of TAMs (14, 36). In addition, CXCL12 and CXCR4 contribute to macrophage recruitment and are downregulated by miR-222 overexpression in TAMs (37). Furthermore, upregulation of the NF-kB p50 transcription factor in TAMs inhibits M1 inflammatory responses (38). We showed that CSF2RA and CD43 were downregulated by miR-7083-5p, suggesting that they play a role in the repolarization of M2 macrophages towards an M1-like phenotype, *via* epigenetic therapy and the miR-7083-5p axis. CSF2RA is involved in the proliferation, differentiation, and activation of hematopoietic cells (27). It is also involved in the progression of triple negative breast cancer cells (39). CD43 is involved in cell adhesion and migration, and is downregulated by the tumor suppressor p53, suggesting its role in tumorigenesis (26, 30, 40). These findings suggest that downregulation of CSF2RA and CD43 is involved in miR7083-5p-mediated M2 to M1 macrophage reprogramming, and contributes to the inhibition of tumor growth. Further studies are needed to identify the downstream pathways of CSF2RA and CD43 involved in macrophage polarization.

Taken together, the combined inhibition of DNA methylation and histone deacetylation may exert anti-tumor growth activity in two ways: it induces epigenetic changes in tumor cells and directly inhibits tumor growth; in contrast, it induces miRNAs that are involved in reprogramming M2-type TAM into an M1-like phenotype, thus paving the way for better immune surveillance in the tumor microenvironment.

## Data availability statement

The original contributions presented in the study are publicly available. This data can be found here: https://www.ncbi.nlm.nih.gov/geo/query/acc.cgi?acc=GSE218160 /GSE218160.

## Ethics statement

The animal study was reviewed and approved by Institutional Animal Care and Use Committee of Kyungpook National University.

## Author contributions

SV performed the experiments and drafted the figures and manuscript. GG and JY performed the animal experiments. T-HK and KH analyzed the miRNA datasets. BL designed the research, analyzed data, and wrote the manuscript. All authors contributed to the article and approved the submitted version.

## Funding

This work was supported by a National Research Foundation grant [2021R1A5A2021614] and the Bio & Medical Technology Development Program [2017M3A9G8083382 and 2020M3A9I4039539] funded by the Ministry of Science and ICT of Korea.

## Acknowledgments

We would like to thank all authors for their contributions to this article.

## Conflict of interest

The authors declare that the research was conducted in the absence of any commercial or financial relationships that could be construed as a potential conflict of interest.

## Publisher’s note

All claims expressed in this article are solely those of the authors and do not necessarily represent those of their affiliated organizations, or those of the publisher, the editors and the reviewers. Any product that may be evaluated in this article, or claim that may be made by its manufacturer, is not guaranteed or endorsed by the publisher.

## Abbreviations

5-aza-dC, 5-aza-2’-deoxycytidine; CM, Conditioned medium; CSF-1, Colony-stimulating factor-1; CSF2RA, Colony-stimulating factor 2 receptor alpha; DMEM, Dulbecco’s modified Eagle’s medium; ELISA, Enzyme-linked immunosorbent assay; FBS, Fetal bovine serum;qRT-PCR, Quantitative reverse transcription-polymerase chain reaction; TAM, Tumor-associated macrophages; TSA, Trichostatin A.

## References

[1] Pollard JW. Tumour-educated macrophages promote tumour progression and metastasis. Nat Rev Cancer (2004) 4:71–8. doi: 10.1038/nrc1256 14708027

[2] Lewis CE Pollard JW. Distinct role of macrophages in different tumor microenvironments. Cancer Res (2006) 66:605–12. doi: 10.1158/0008-5472.CAN-05-4005 16423985

[3] Noy R Pollard JW. Tumor-associated macrophages: from mechanisms to therapy. Immunity. (2014) 41:49–61. doi: 10.1016/j.immuni.2014.06.010 25035953 PMC4137410

[4] Colegio OR Chu N-Q Szabo AL Chu T Rhebergen AM Jairam V. Functional polarization of tumour-associated macrophages by tumour-derived lactic acid. Nature. (2014) 513:559–63. doi: 10.1038/nature13490 PMC4301845 25043024

[5] Kusmartsev S Gabrilovich DI. STAT1 signaling regulates tumor-associated macrophage-mediated T cell deletion. J Immunol (2005) 174:4880–91. doi: 10.4049/jimmunol.174.8.4880 15814715

[6] Ivashkiv LB. Epigenetic regulation of macrophage polarization and function. Trends Immunol (2013) 34:216–23. doi: 10.1016/j.it.2012.11.001 PMC3647003 23218730

[7] Alvarez-Errico D Vento-Tormo R Sieweke M Ballestar E. Epigenetic control of myeloid cell differentiation, identity and function. Nat Rev Immunol (2015) 15:7–17. doi: 10.1038/nri3777 25534619

[8] Ishii M Wen H Corsa CA Liu T Coelho AL Allen RM. Epigenetic regulation of the alternatively activated macrophage phenotype. Blood. (2009) 114:3244–54. doi: 10.1182/blood-2009-04-217620 PMC2759649 19567879

[9] Satoh T Takeuchi O Vandenbon A Yasuda K Tanaka Y Kumagai Y. The Jmjd3-Irf4 axis regulates M2 macrophage polarization and host responses against helminth infection. Nat Immunol (2010) 11:936–44. doi: 10.1038/ni.1920 20729857

[10] Serrat N Pereira-Lopes S Comalada M Lloberas J Celada A. Deacetylation of C/EBPβ is required for IL-4-induced arginase-1 expression in murine macrophages. Eur J Immunol (2012) 42:3028–37. doi: 10.1002/eji.201242413 22865229

[11] Li X Su X Liu R Pan Y Fang J Cao L. HDAC inhibition potentiates anti-tumor activity of macrophages and enhances anti-PD-L1-mediated tumor suppression. Oncogene. (2021) 40:1836–50. doi: 10.1038/s41388-020-01636-x PMC7946638 33564072

[12] de Groot AE Pienta KJ. Epigenetic control of macrophage polarization: implications for targeting tumor-associated macrophages. Oncotarget. (2018) 9:20908–27. doi: 10.18632/oncotarget.24556 PMC5945509 29755698

[13] Shi R Zhao K Wang T Yuan J Zhang D Xiang W. 5-aza-2′-deoxycytidine potentiates anti-tumor immunity in colorectal peritoneal metastasis by modulating ABC A9-mediated cholesterol accumulation in macrophages. Theranostics. (2022) 12:875. doi: 10.7150/thno.66420 34976218 PMC8692916

[14] Curtale G Rubino M Locati M. MicroRNAs as molecular switches in macrophage activation. Front Immunol (2019) 10:799. doi: 10.3389/fimmu.2019.00799 31057539 PMC6478758

[15] Zonari E Pucci F Saini M Mazzieri R Politi LS Gentner B. A role for miR-155 in enabling tumor-infiltrating innate immune cells to mount effective antitumor responses in mice. Blood. (2013) 122:243–52. doi: 10.1182/blood-2012-08-449306 23487026

[16] Ali SR Humphreys KJ McKinnon RA Michael MZ. Impact of histone deacetylase inhibitors on microRNA expression and cancer therapy: A review. Drug Dev Res (2015) 76:296–317. doi: 10.1002/ddr.21268 26303212

[17] Squadrito ML Pucci F Magri L Moi D Gilfillan GD Ranghetti A. miR-511-3p modulates genetic programs of tumor-associated macrophages. Cell Rep (2012) 1:141–54. doi: 10.1016/j.celrep.2011.12.005 22832163

[18] Osella M Riba A Testori A Corà D Caselle M. Interplay of microRNA and epigenetic regulation in the human regulatory network. Front Genet (2014) 5:345. doi: 10.3389/fgene.2014.00345 25339974 PMC4186481

[19] Zhao ZN Bai JX Zhou Q Yan B Qin WW Jia LT. TSA suppresses miR-106b-93-25 cluster expression through downregulation of MYC and inhibits proliferation and induces apoptosis in human EMC. PloS One (2012) 7:e45133. doi: 10.1371/journal.pone.0045133 23028803 PMC3446970

[20] Kalinowski FC Brown RA Ganda C Giles KM Epis MR Horsham J. microRNA-7: a tumor suppressor miRNA with therapeutic potential. Int J Biochem Cell Biol (2014) 54:312–7. doi: 10.1016/j.biocel.2014.05.040 24907395

[21] Zhang B Wang J Gao J Guo Y Chen X Wang B. Alternatively activated RAW264. 7 macrophages enhance tumor lymphangiogenesis in mouse lung adenocarcinoma. J Cell Biochem (2009) 107:134–43. doi: 10.1002/jcb.22110 19241443

[22] Gunassekaran GR Poongkavithai Vadevoo SM Baek MC Lee B. M1 macrophage exosomes engineered to foster M1 polarization and target the IL-4 receptor inhibit tumor growth by reprogramming tumor-associated macrophages into M1-like macrophages. Biomaterials. (2021) 278:121137. doi: 10.1016/j.biomaterials.2021.121137 34560422

[23] Livak KJ Schmittgen TD. Analysis of relative gene expression data using real-time quantitative PCR and the 2– ΔΔCT method. methods. (2001) 25:402–8. doi: 10.1006/meth.2001.1262 11846609

[24] Huang DW Sherman BT Lempicki RA. Systematic and integrative analysis of large gene lists using DAVID bioinformatics resources. Nat Protoc (2009) 4:44–57. doi: 10.1038/nprot.2008.211 19131956

[25] Uhlen M Oksvold P Fagerberg L Lundberg E Jonasson K Forsberg M. Towards a knowledge-based human protein atlas. Nat Biotechnol (2010) 28:1248–50. doi: 10.1038/nbt1210-1248 21139605

[26] Baeckström D Zhang K Asker N Rüetschi U Ek M Hansson GC. Expression of the leukocyte-associated sialoglycoprotein CD43 by a colon carcinoma cell line. J Biol Chem (1995) 270:13688–92. doi: 10.1074/jbc.270.23.13688 7775421

[27] Hercus TR Broughton SE Ekert PG Ramshaw HS Perugini M Grimbaldeston M. The GM-CSF receptor family: mechanism of activation and implications for disease. Growth factors (2012) 30:63–75. doi: 10.3109/08977194.2011.649919 22257375

[28] Huang X Hu P Zhang J. Genomic analysis of the prognostic value of colony-stimulating factors (CSFs) and colony-stimulating factor receptors (CSFRs) across 24 solid cancer types. Ann Transl Med (2020) 8:994. doi: 10.21037/atm-20-5363 PMC7475477 32953794

[29] Ostberg JR Barth RK Frelinger JG. The Roman god janus: a paradigm for the function of CD43. Immunol Today (1998) 19:546–50. doi: 10.1016/s0167-5699(98)01343-7 9864944

[30] Tuccillo FM De Laurentiis A Palmieri C Fiume G Bonelli P Borrelli A. Aberrant glycosylation as biomarker for cancer: focus on CD43. BioMed Res Int (2014) 2014:742831. doi: 10.1155/2014/742831 PMC3943294 24689054

[31] Waghray M Yalamanchili M Dziubinski M Zeinali M Erkkinen M Yang H. GM-CSF mediates mesenchymal–epithelial cross-talk in pancreatic cancer. Cancer Discovery (2016) 6:886–99. doi: 10.1158/2159-8290.CD-15-0947 PMC5549011 27184426

[32] Saito Y Liang G Egger G Friedman JM Chuang JC Coetzee GA. Specific activation of microRNA-127 with downregulation of the proto-oncogene BCL6 by chromatin-modifying drugs in human cancer cells. Cancer Cell (2006) 9:435–43. doi: 10.1016/j.ccr.2006.04.020 16766263

[33] Lu Z Zou J Li S Topper MJ Tao Y Zhang H. Epigenetic therapy inhibits metastases by disrupting premetastatic niches. Nature. (2020) 579:284–90. doi: 10.1038/s41586-020-2054-x PMC8765085 32103175

[34] Lin EY Nguyen AV Russell RG Pollard JW. Colony-stimulating factor 1 promotes progression of mammary tumors to malignancy. J Exp Med (2001) 193:727–40. doi: 10.1084/jem.193.6.727 PMC2193412 11257139

[35] Zabuawala T Taffany DA Sharma SM Merchant A Adair B Srinivasan R. An ets2-driven transcriptional program in tumor-associated macrophages promotes tumor metastasis. Cancer Res (2010) 70:1323–33. doi: 10.1158/0008-5472.CAN-09-1474 PMC2822898 20145133

[36] Mathsyaraja H Thies K Taffany DA Deighan C Liu T Yu L. CSF1-ETS2-induced microRNA in myeloid cells promote metastatic tumor growth. Oncogene. (2015) 34:3651–61. doi: 10.1038/onc.2014.294 PMC4369473 25241894

[37] Li Y Zhao L Shi B Ma S Xu Z Ge Y. Functions of miR-146a and miR-222 in tumor-associated macrophages in breast cancer. Sci Rep (2015) 5:18648. doi: 10.1038/srep18648 26689540 PMC4686897

[38] Saccani A Schioppa T Porta C Biswas SK Nebuloni M Vago L. p50 nuclear factor-kappaB overexpression in tumor-associated macrophages inhibits M1 inflammatory responses and antitumor resistance. Cancer Res (2006) 66:11432–40. doi: 10.1158/0008-5472.CAN-06-1867 17145890

[39] Karagoz K Sinha R Arga KY. Triple negative breast cancer: a multi-omics network discovery strategy for candidate targets and driving pathways. Omics J Integr Biol (2015) 19:115–30. doi: 10.1089/omi.2014.0135 25611337

[40] Kadaja-Saarepuu L Lõoke M Balikova A Maimets T. Tumor suppressor p53 down-regulates expression of human leukocyte marker CD43 in non-hematopoietic tumor cells. Int J Oncol (2012) 40:567. doi: 10.3892/ijo.2011.1208 21947346

