# Supplementary material for: Epigenetic therapy reprograms M2-type tumor-associated macrophages into an M1-like phenotype by upregulating miR-7083-5p
Source: Front Immunol. 2022 Nov 22;13:976196. doi: 10.3389/fimmu.2022.976196 (PMC9724234; doi:10.3389/fimmu.2022.976196)
Supplement: Supplementary file 1 [file DataSheet_1.docx]

Supplementary Materials

**Supplementary Tables**

**Supplementary Table 1. miRNAs upregulated and downregulated in M2-type macrophages after treatment with the combination of AZA and TSA, as compared to those in untreated M2-type macrophages.**

| Upregulated (>3-fold) | | Downregulated (>3-fold) | |
| --- | --- | --- | --- |
| mmu-miR-7083-5p  mmu-miR-7043-5p  mmu-miR-6240  mmu-miR-3473f  mmu-miR-7035-5p  mmu-miR-184-3p  mmu-miR-25-5p  mmu-miR-7044-5p  mmu-miR-8117  mmu-miR-6937-5p  mmu-miR-696  mmu-miR-467e-5p  mmu-miR-6970-5p  mmu-miR-7047-5p  mmu-miR-466d-3p  mmu-miR-3473g  mmu-let-7e-3p  mmu-miR-3473e | 36.0  7.7  5.8  5.6  5.3  4.9  4.6  4.5  4.4  4.3  3.8  3.3  3.3  3.3  3.1  3.1  3.1  3.1 | mmu-miR-214-3p  mmu-miR-149-5p  mmu-miR-130a-3p  mmu-miR-199b-3p  mmu-miR-199a-3p  mmu-miR-3472  mmu-miR-23b-5p  mmu-miR-30e-3p  mmu-miR-27b-3p  mmu-miR-19a-3p  mmu-miR-27b-5p  -  -  -  -  -  -  - | 5.3  5.0  4.6  4.6  4.6  4.3  4.3  4.2  4.1  4.1  4.0  -  -  -  -  -  -  - |
|  |  |  |  |

**Supplementary Table 2. miRNAs upregulated and downregulated in M1-type macrophages, as compared to those in untreated M2-type macrophages.**

| Upregulated (>3-fold) | | Downregulated (>3-fold) | |
| --- | --- | --- | --- |
| mmu-miR-184-3p  mmu-miR-7043-5p  mmu-miR-125a-3p  mmu-miR-31-5p  mmu-miR-6240  mmu-miR-7044-5p  mmu-miR-3473f  mmu-miR-714  mmu-miR-21a-3p | 14.6  7.5  4.5  4.4  4.3  3.9  3.2  3.2  3.1 | mmu-miR-149-5p  mmu-miR-511-3p  mmu-miR-1839-3p  mmu-miR-27b-3p  mmu-miR-30a-3p  mmu-miR-322-3p  -  -  - | 8.1  5.9  5.4  5.2  4.7  4.7  -  -  - |

**Supplementary Table 3. TargetScan analysis of candidate target genes of miR-7083-5p.**

| Gene Symbol | Gene Description |
| --- | --- |
| *acadm* | Acyl-coenzyme A dehydrogenase, medium chain |
| *ankrd52* | Ankyrin repeat domain 52 |
| *bend3* | BEN domain containing 3 |
| *cd43* | CD43 or sialophorin |
| *cnnm3* | Cyclin M3 |
| *crybg3* | Beta-gamma crystallin domain containing 3 |
| *csde1* | Cold shock domain containing E1, RNA-binding |
| *csf2ra* | Colony-stimulating factor 2 receptor, subunit alpha |
| *dio2* | Deiodinase, iodothyronine, type II |
| *dmpk* | Dystrophia myotonica-protein kinase |
| *dnali1* | Dynein, axonemal, light intermediate polypeptide 1 |
| *gabpb2* | GA repeat-binding protein, beta 2 |
| *grin1* | Glutamate receptor, ionotropic, NMDA1 (zeta 1) |
| *kbtbd11* | Kelch repeat and BTB (POZ) domain containing 11 |
| *lypla2* | Lysophospholipase 2 |
| *lrrc23* | Leucine-rich repeat containing 23 |
| *man1a2* | Mannosidase, alpha, class 1A, member 2 |
| *mprip* | Myosin phosphatase Rho-interacting protein |
| *mtss1l* | Metastasis suppressor 1-like |
| *myh10* | Myosin, heavy polypeptide 10, non-muscle |
| *parp3* | Poly (ADP-ribose) polymerase family, member 3 |
| *satb1* | Special AT-rich sequence-binding protein 1 |
| *sgk3* | Serum/glucocorticoid regulated kinase 3 |
| *slc25a46* | Solute carrier family 25, member 46 |
| *srrm4* | Serine/arginine repetitive matrix 4 |
| *sowahc* | Sosondowah ankyrin repeat domain family member C |
| *sypl* | synaptophysin-like protein |
| *ssr3* | Signal sequence receptor, gamma |
| *tas1r3* | Taste receptor, type 1, member 3 |
| *vwa7* | von Willebrand factor A domain containing 7 |
| *wdr77* | WD repeat domain 77 |
| *zfp426* | Zinc finger protein 426 |

**Supplementary Figures**

**
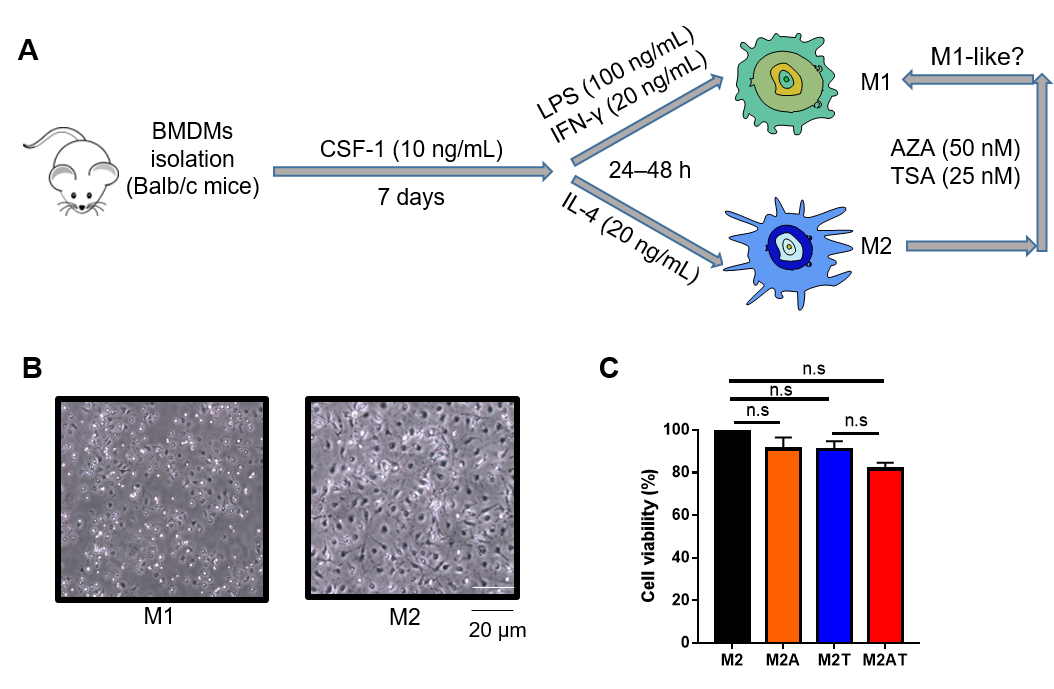
**

**Supplementary Figure 1.** **Polarization of M1- and M2-type macrophages and viability of M2-type macrophages after treatment with AZA and TSA. (A)** Schemes of the preparation and polarization of M1- and M2-type macrophages from BMDMs. Bone marrow cells were cultured in DMEM supplemented with 10 ng/mL of colony-stimulating factor-1 (CSF-1) and 10% FBS for 7 d. The culture medium was changed every other day. After the CSF-1 treatment, BMDMs were incubated with 100 ng/mL of lipopolysaccharide (LPS) and 20 ng/mL of recombinant mouse IFN-γ for 24–48 h to polarize them into M1 macrophages. For M2-polarization, BMDMs were incubated with 20 ng/mL of recombinant mouse IL-4 for 24–48 h. M2-polarized macrophages were treated with either 50 nM AZA for 72 h or 25 nM TSA for 48 h. For combined treatment, the cells were treated with AZA for 24 h followed by treatment with AZA and TSA for 48 h. (**B)** Representative images of M1- and M2-polarized macrophages in the cultures. (**C)** Viability of M2-macrophages (M2) after treatment with AZA (M2A) and TSA (M2T), alone or in combination (M2AT). Data are presented as the mean ± S.D. of triplicates. n.s., not significant, as assessed using one-way ANOVA.


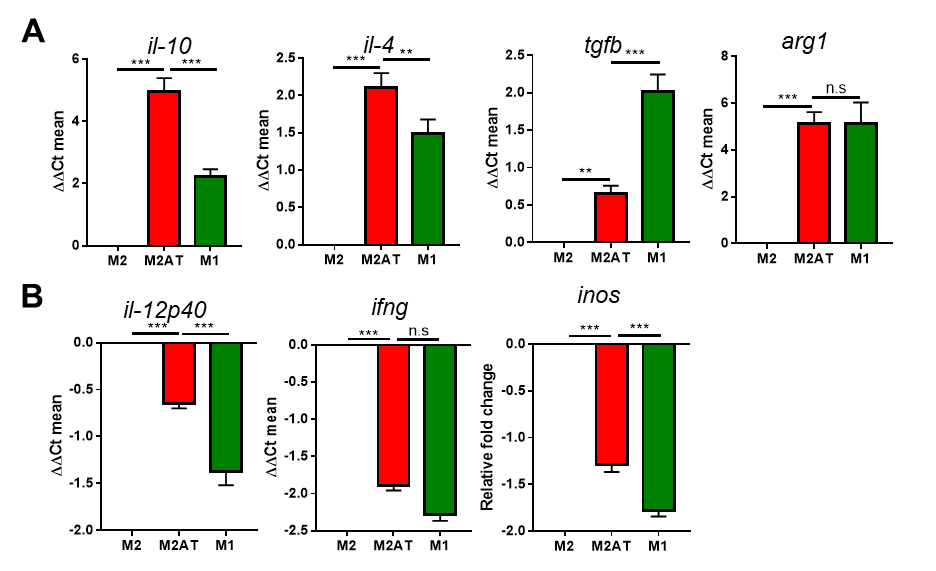


**Supplementary Figure 2. ΔΔCt values of the mRNA levels in M2 macrophages after epigenetic therapy.** qRT-PCR analysis of the mRNA levels of M2-type (A) and M1-type (B) cytokines and markers after treatment of M2-type macrophages with 5-aza-dC and TSA in combination (M2AT). Data represent the mean ± SD of three separate experiments performed in triplicates. **, *P*<0.01; ***, *P*<0.001; n.s., not significant, as assessed using one-way ANOVA followed by Tukey’s multiple comparison *post-hoc* test.

**
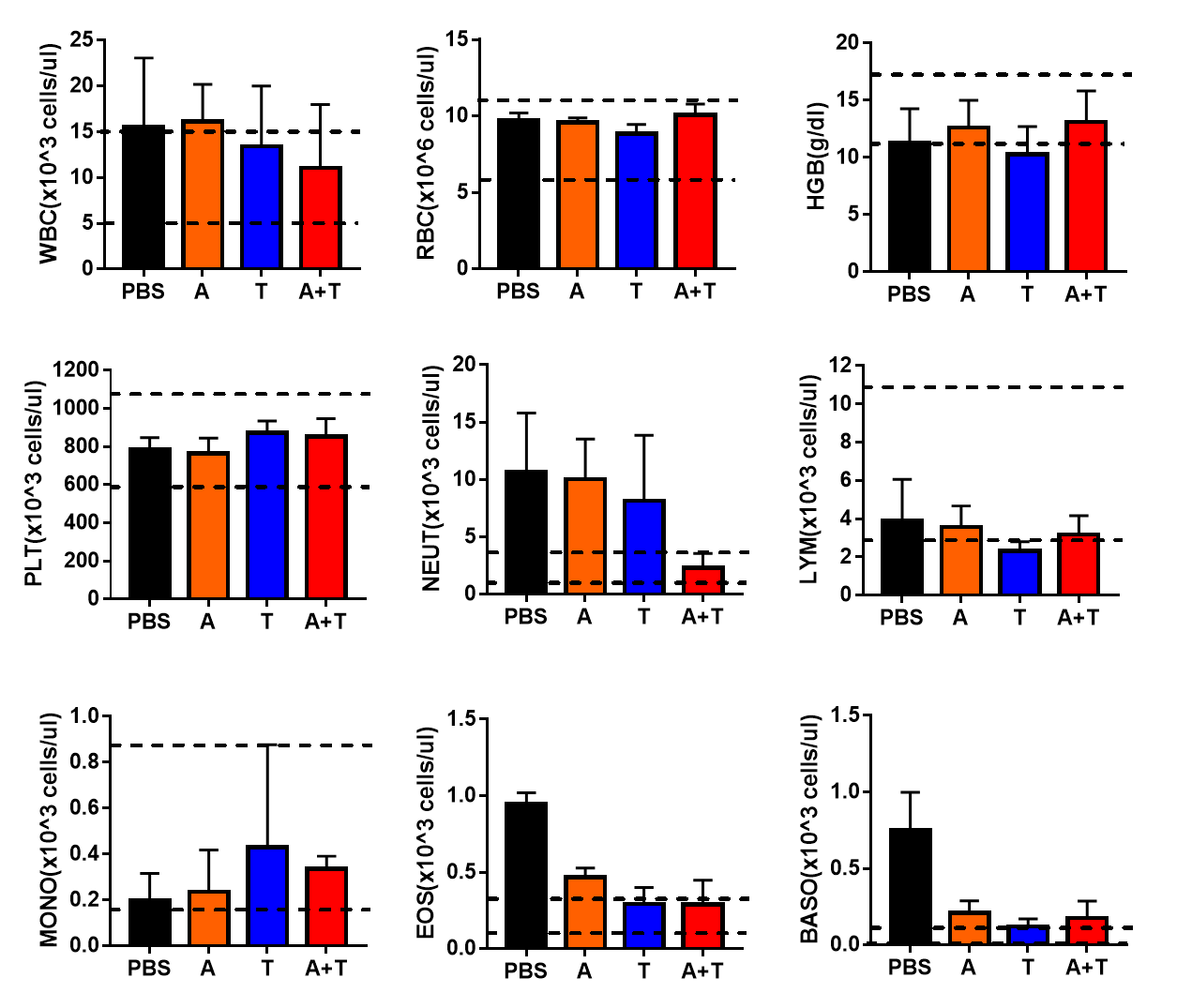
**

**Supplementary Figure 3. Analysis of hematologic parameters after epigenetic therapy.** Mice bearing 4T1 or 4T1-luc tumors (approximately 100–150 mm^3^ in size) in the left and lower mammary gland were treated with 5-aza-dC (referred as A) and TSA (referred as T), alone or in combination (1 mg/kg body weight 5-aza-dC and 0.3 mg/kg body weight TSA, once a day, for 5 d), at 2 weeks after tumor inoculation. After treatments, blood was collected and analyzed for the hematologic parameters. Dotted lines represent normal values of each parameter.

**
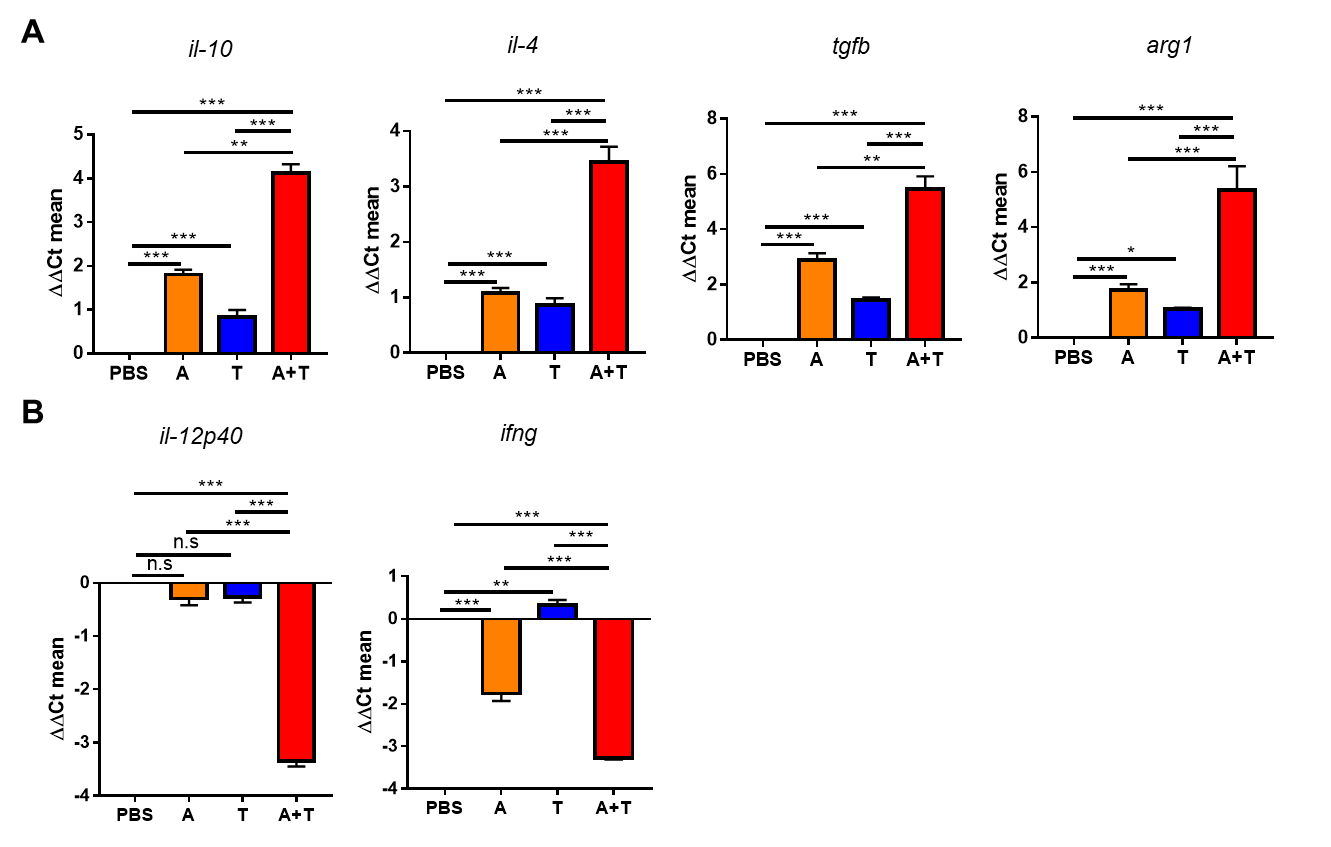
**

**Supplementary Figure 4. ΔΔCt values of the mRNA levels** **in tumor tissues after epigenetic therapy.** qRT-PCR analysis of the relative mRNA levels of M2- (A) and M1-type (B) cytokines and markers in tumor tissues after treatment of 4T1 tumor-bearing mice with 5-aza-dC (referred as A) and TSA (referred as T), alone or in combination (A+T). Data represent the mean ± SD. **, *P*<0.01; ***, *P*<0.001; n.s., not significant (*n*=3 mice per group), as assessed using one-way ANOVA followed by Tukey’s multiple comparison *post-hoc* test.

**
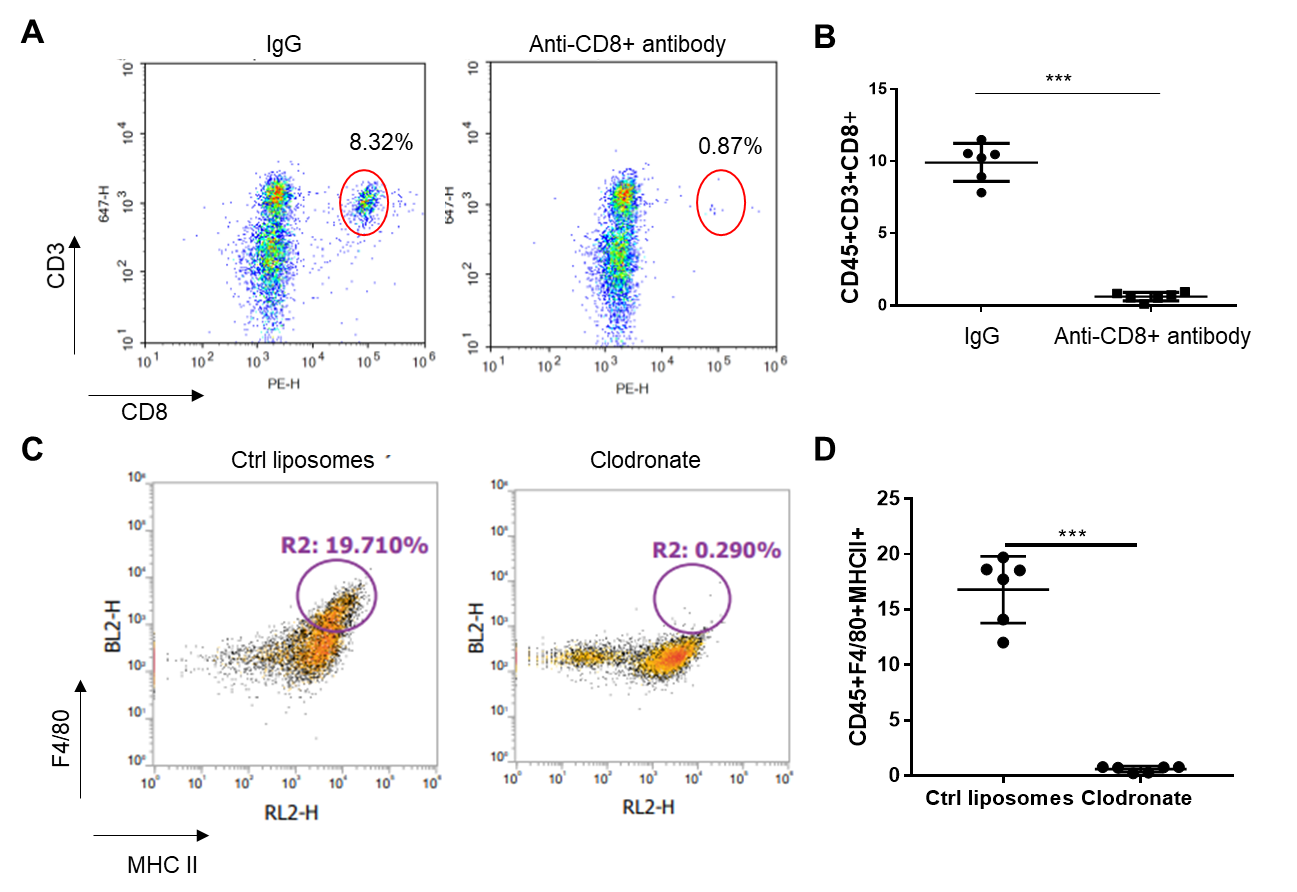
**

**Supplementary Figure 5. Depletion of CD8^+^ T cells and macrophages in tumor-bearing mice. (A)** A representative flow cytometric analysis of splenocytes isolated from a tumor-bearing mouse after treatment with either IgG control or anti-CD8 antibody. Balb/c mice were intraperitoneally injected with an anti-CD8 neutralizing antibody one day before inoculation of 4T1 tumor cells and every 5 day thereafter. (**B)** Quantification of the population of CD8**^+^** T cells after treatments. **(C)** A representative flow cytometric analysis of splenocytes isolated from a tumor-bearing mouse after treatment with either control (Ctrl) liposomes or clodronate liposomes. Mice bearing 4T1 tumor were intraperitoneally injected with clodronate liposomes 2 days prior to start of treatment. **(D)** Quantification of the population of macrophages after treatments. Data are presented as the mean ± SD of six replicates. Experiments were performed in three mice, with similar results. ***, *P*<0.001, as assessed using one-way ANOVA followed by Tukey’s multiple comparison *post-hoc* test.

**
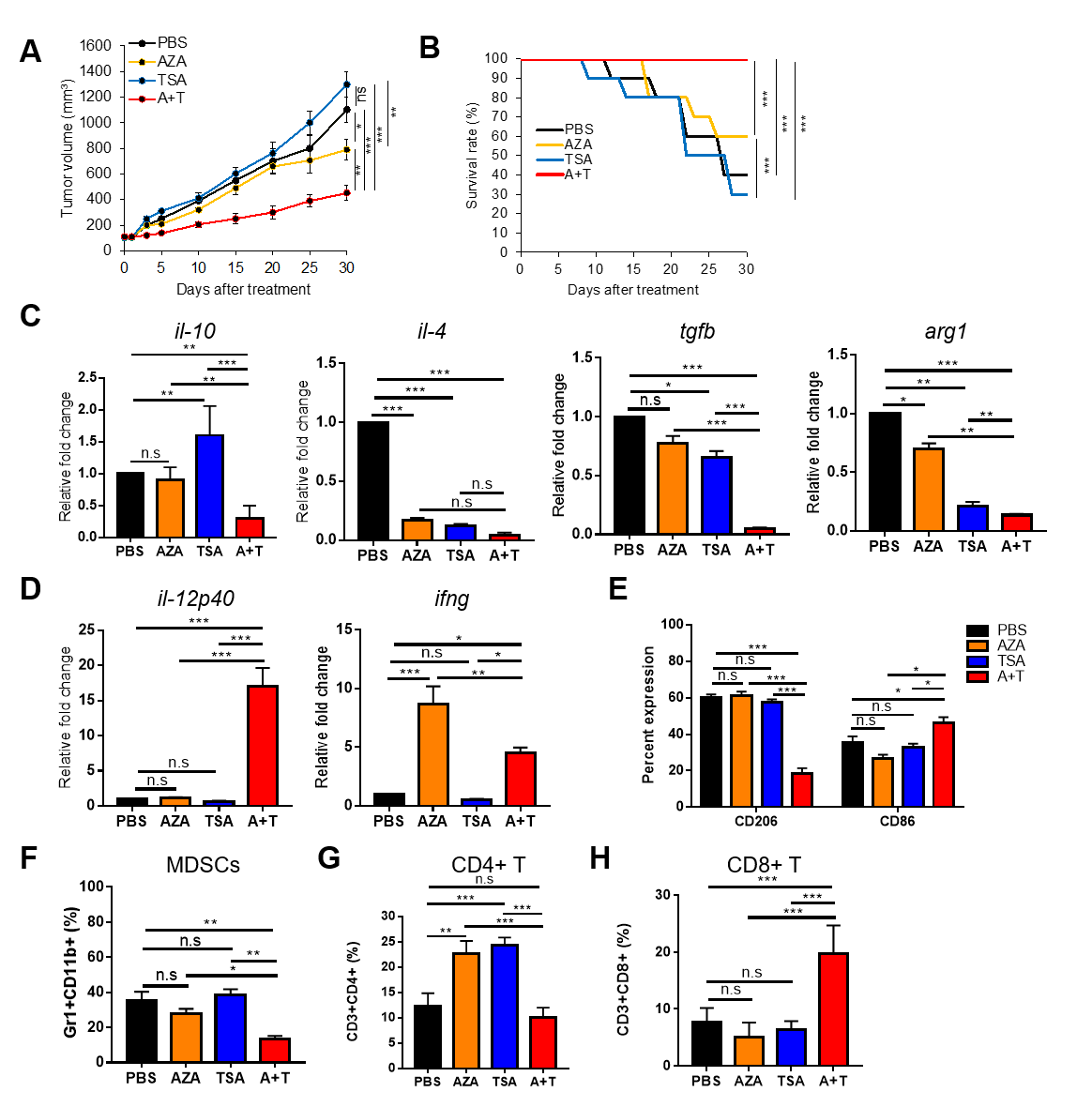
**

**Supplementary Figure 6. Epigenetic therapy upon treatment AZA and TSA synergistically inhibits lung tumor growth in mice.** Mice bearing LLC lung tumor (approximately 100–150 mm^3^ in size) were treated with AZA and TSA, alone or in combination (A+T,1 mg/kg body weight AZA and 0.3 mg/kg body weight TSA, once a day, for 5 d). **(A-B)** Tumor volumes (A) and survival rates (B) of the mice after treatments. (**C-D**) qRT-PCR analysis of the relative mRNA levels of M2-type (C) and M1-type (D) cytokines and markers in the tumor tissues after treatments. (**E**–**H)** Flow cytometric analysis of the population of CD206**^+^** M2 macrophages and CD86**^+^** M1 macrophages (E), Gr1**^+^**CD11b**^+^** MDSCs (F), CD3**^+^**CD4**^+^** T cells (G), and CD3**^+^**CD8**^+^** T cells (H) in tumor tissues after treatments. *, *P*<0.05; **, *P*<0.01; ***, *P*<0.001; n.s., not significant (*n*=10 per group), as assessed using one-way ANOVA.


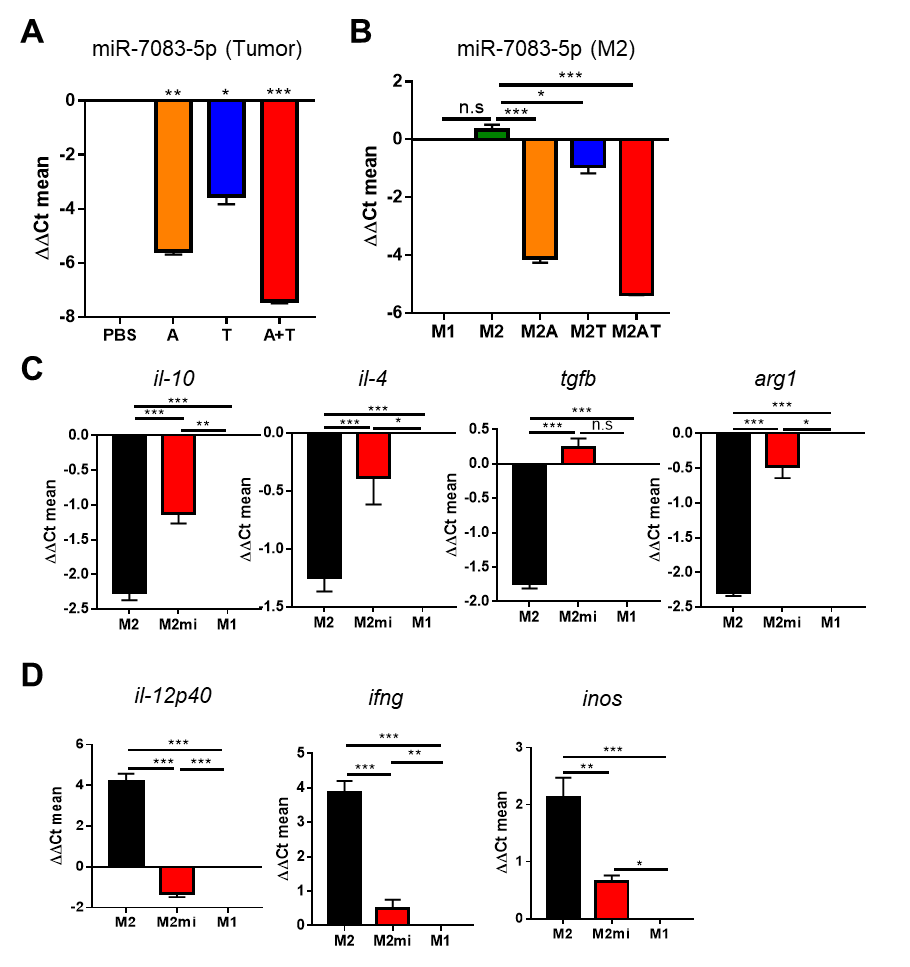


**Supplementary Figure 7. ΔΔCt values of the mRNA levels** **in M2 macrophages after treatment with miR-7083-5p. (A-B)** qRT-PCR analysis of the relative levels of miR-7083-5p in 4T1 tumor tissues, after treatments with 5-aza-dC (referred as A) and TSA (referred as T), alone or in combination (A+T), (C) and in M2 macrophages, after treatments with 5-aza-dC (M2A) and TSA (M2T), alone or in combination (D). **(C-D)** qRT-PCR analysis of the relative mRNA levels of M2- (C) and M1-type (D) cytokines and markers in M2 macrophages after transfection of miR-7083-5p (M2mi).


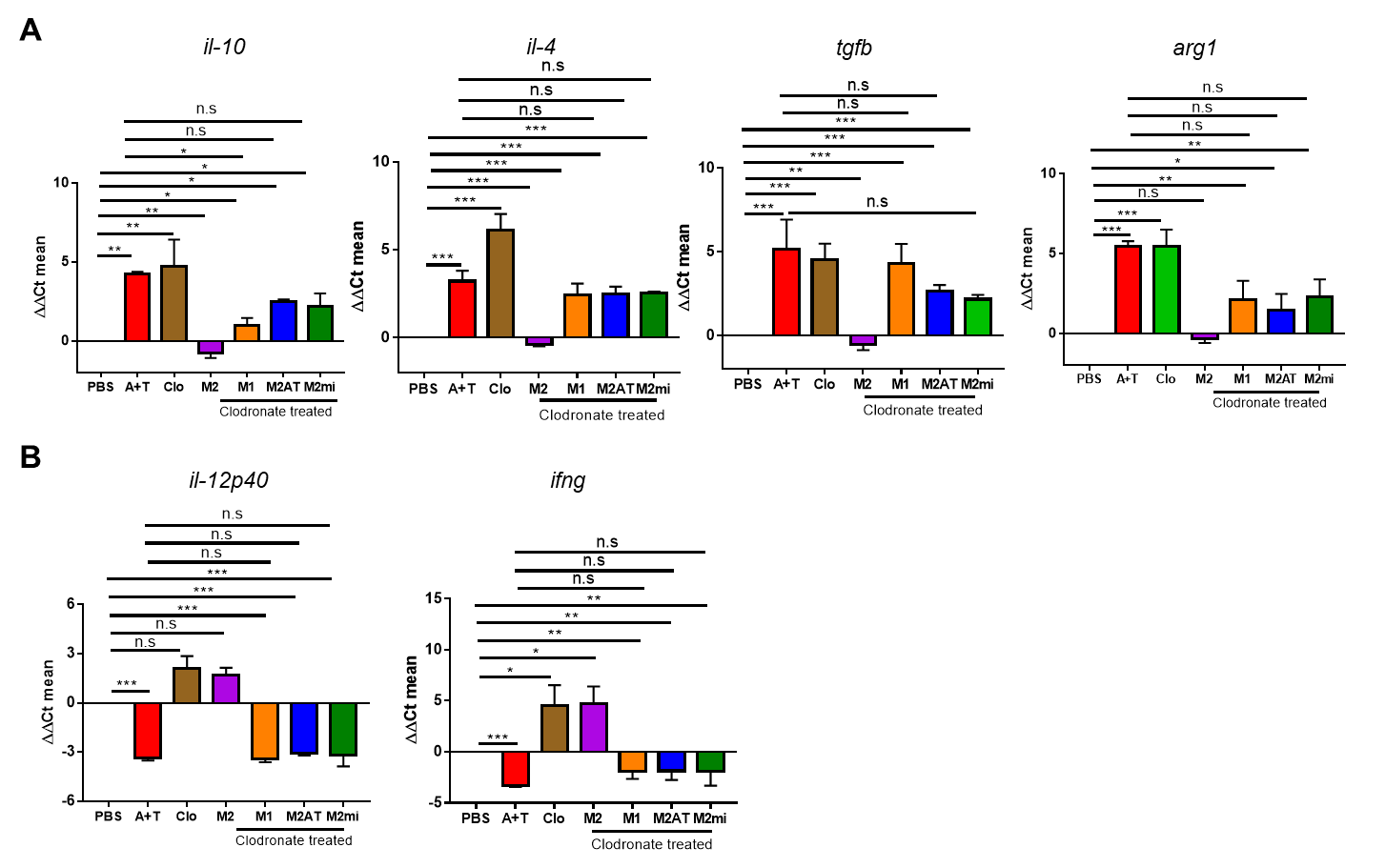


**Supplementary Figure 8. ΔΔCt values of the mRNA levels** **in tumor tissues after adoptive transfer of M2 macrophages.** qRT-PCR analysis of the relative mRNA levels of M2- (A) and M1-type (B) cytokines and markers in tumor tissues after treatment of 4T1 tumor-bearing mice with clodronate and subsequent adoptive transfer of M2 macrophages pre-treated with miR-7083-5p (M2mi) or combination of 5-aza-dC and TSA (M2AT). M1 and M2 macrophages were included as control. A+T, combined treatment with 5-aza-dC and TSA. Clo, clodronate. Data represent the mean ± SD. *, *P*<0.05; **, *P*<0.01; ***, *P*<0.001; n.s., not significant (*n*=3 mice per group), as assessed using one-way ANOVA followed by Tukey’s multiple comparison *post-hoc* test.


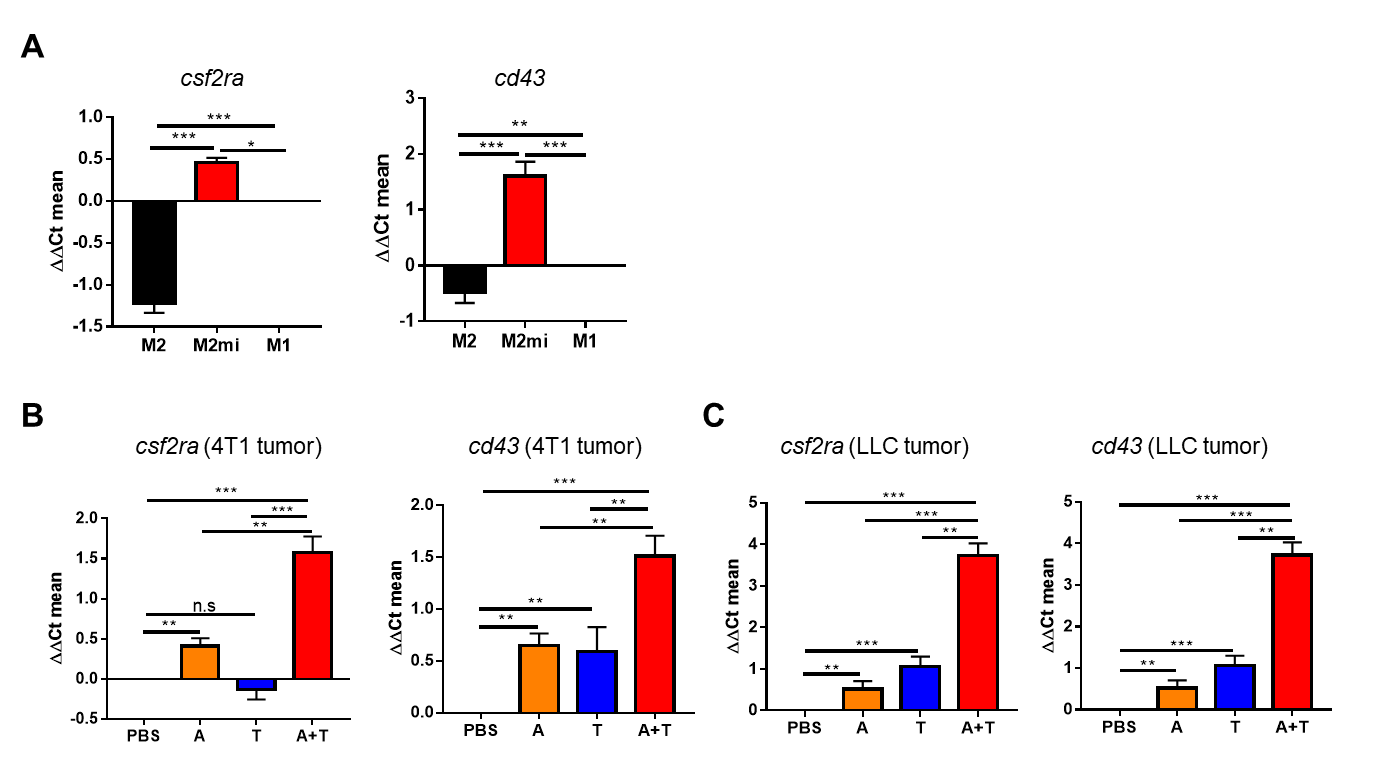


**Supplementary Figure 9. ΔΔCt values of the mRNA levels** **in M2 macrophages and tumor tissues after epigenetic therapy. (A)** qRT-PCR analysis of relative mRNA levels of *csf2ra* and *cd43* in M2 macrophages after transfection with miR-7083-5p (M2mi). **(B-C)** qRT-PCR analysis of the relative mRNA levels of *csf2ra* and *cd43* in 4T1 (B) and LLC (C) tumor tissues after treatments with 5-aza-dC (referred as A) and TSA (referred as T), alone or in combination (A+T). Data have been presented as the mean ± S.D. of three separate experiments performed in five replicates. *, *P*<0.05; **, *P*<0.01; ***, *P*<0.001; n.s., not significant, as assessed using one-way ANOVA followed by Tukey’s multiple comparison *post-hoc* test.
